# Quantitative Oculomotor Assessment in Hereditary Ataxia: Discriminatory Power, Correlation with Severity Measures, and Recommended Parameters for Specific Genotypes

**DOI:** 10.1007/s12311-023-01514-8

**Published:** 2023-01-14

**Authors:** Pilar Garces, Chrystalina A. Antoniades, Anna Sobanska, Norbert Kovacs, Sarah H. Ying, Anoopum S. Gupta, Susan Perlman, David J. Szmulewicz, Chiara Pane, Andrea H. Németh, Laura B. Jardim, Giulia Coarelli, Michaela Dankova, Andreas Traschütz, Alexander A. Tarnutzer

**Affiliations:** 1 Roche Pharma Research and Early Development, Neuroscience and Rare Diseases, Roche Innovation Center Basel, Basel, Switzerland; 2 NeuroMetrology Lab, Nuffield Department of Clinical Neurosciences, Medical Sciences Division, University of Oxford, Oxford, OX3 9DU UK; 3 Department of Clinical Neurophysiology, Institute of Psychiatry and Neurology, Warsaw, Poland; 4 Department of Neurology, Medical School, University of Pecs, Pecs, Hungary; 5 Department of Otology and Laryngology and Department of Neurology, Harvard Medical School, Boston, MA USA; 6 Department of Neurology, Massachusetts General Hospital, Harvard Medical School, Boston, MA USA; 7 University of California Los Angeles, Los Angeles, CA USA; 8 Balance Disorders and Ataxia Service, Royal Victoria Eye and Ear Hospital, East Melbourne, Melbourne, VIC 3002 Australia; 9 The Florey Institute of Neuroscience and Mental Health, Parkville, Melbourne, VIC 3052 Australia; 10 Department of Neurosciences and Reproductive and Odontostomatological Sciences, University of Naples “Federico II”, Naples, Italy; 11 Nuffield Department of Clinical Neurosciences, University of Oxford, Oxford, UK; 12 Oxford Centre for Genomic Medicine, Oxford University Hospitals NHS Trust, Oxford, UK; 13 Departamento de Medicina Interna, Universidade Federal do Rio Grande do Sul, Porto Alegre, Brazil; 14 Serviço de Genética Médica/Centro de Pesquisa Clínica e Experimental, Hospital de Clínicas de Porto Alegre, Porto Alegre, Brazil; 15 Institut du Cerveau-Paris Brain Institute-ICM, Inserm U1127, CNRS UMR7225, Sorbonne Université, Paris, France; 16 Department of Genetics, Neurogene National Reference Centre for Rare Diseases, Pitié-Salpêtrière University Hospital, Assistance Publique, Hôpitaux de Paris, Paris, France; 17 Department of Neurology, Centre of Hereditary Ataxias, 2nd Faculty of Medicine, Charles University and Motol University Hospital, Prague, Czech Republic; 18 Research Division “Translational Genomics of Neurodegenerative Diseases,” Hertie-Institute for Clinical Brain Research and Center of Neurology, University of Tübingen, Tübingen, Germany; 19 German Center for Neurodegenerative Diseases (DZNE), University of Tübingen, Tübingen, Germany; 20 Cantonal Hospital of Baden, Baden, Switzerland; 21 Faculty of Medicine, University of Zurich, Zurich, Switzerland

**Keywords:** Oculomotor, Vestibular, Eye movement recordings, Hereditary ataxia, Systematic review, Recommendations

## Abstract

Characterizing bedside oculomotor deficits is a critical factor in defining the clinical presentation of hereditary ataxias. Quantitative assessments are increasingly available and have significant advantages, including comparability over time, reduced examiner dependency, and sensitivity to subtle changes. To delineate the potential of quantitative oculomotor assessments as digital-motor outcome measures for clinical trials in ataxia, we searched MEDLINE for articles reporting on quantitative eye movement recordings in genetically confirmed or suspected hereditary ataxias, asking which paradigms are most promising for capturing disease progression and treatment response. Eighty-nine manuscripts identified reported on 1541 patients, including spinocerebellar ataxias (SCA2, *n* = 421), SCA3 (*n* = 268), SCA6 (*n* = 117), other SCAs (*n* = 97), Friedreich ataxia (FRDA, *n* = 178), Niemann-Pick disease type C (NPC, *n* = 57), and ataxia-telangiectasia (*n* = 85) as largest cohorts. Whereas most studies reported discriminatory power of oculomotor assessments in diagnostics, few explored their value for monitoring genotype-specific disease progression (*n* = 2; SCA2) or treatment response (*n* = 8; SCA2, FRDA, NPC, ataxia-telangiectasia, episodic-ataxia 4). Oculomotor parameters correlated with disease severity measures including clinical scores (*n* = 18 studies (SARA: *n* = 9)), chronological measures (e.g., age, disease duration, time-to-symptom onset; *n* = 17), genetic stratification (*n* = 9), and imaging measures of atrophy (*n* = 5). Recurrent correlations across many ataxias (SCA2/3/17, FRDA, NPC) suggest saccadic eye movements as potentially generic quantitative oculomotor outcome. Recommendation of other paradigms was limited by the scarcity of cross-validating correlations, except saccadic intrusions (FRDA), pursuit eye movements (SCA17), and quantitative head-impulse testing (SCA3/6). This work aids in understanding the current knowledge of quantitative oculomotor parameters in hereditary ataxias, and identifies gaps for validation as potential trial outcome measures in specific ataxia genotypes.

**Supplementary Information:**

The online version contains supplementary material available at 10.1007/s12311-023-01514-8.

## Introduction

Eye movement abnormalities are common in hereditary ataxia [1–3]. The characterization of oculomotor and vestibular deficits has contributed significantly to disease and gene discovery by contributing to the delineation of novel disease phenotypes, which has in turn informed our understanding of the clinical presentations of specific hereditary ataxias [4–7]. While oculomotor testing may be performed through expert visual interpretation at the bedside, objective and quantitative assessments have significant advantages, including comparability over time, reduced examiner dependency, and inter-/intra-individual variability along with increased sensitivity. Accordingly, quantitative oculomotor assessments have been sensitive enough to capture within-subject longitudinal disease progression and early signs of efficacy for future interventions [8] and alterations in at-risk carriers at the pre-ataxia stage [9] and to distinguish between ataxia diagnoses [10]. Together with the increasing availability of video-oculographic (VOG) devices for multicenter applicability, this sensitivity of quantitative oculomotor assessments makes them promising digital motor biomarkers for emerging treatment trials in ataxia.

Among the broad range of oculomotor alterations in ataxia, some are generic for cerebellar ataxia (e.g., slow saccades) [11], while others are more specific for particular hereditary ataxias (e.g., an impaired vestibulo-ocular reflex in RFC1-ataxia [12] or SCA6 [13]). An increasing number of studies are investigating quantitative oculomotor parameters in ataxia, and their findings have been partially reviewed at the level of individual genetic ataxias [14], or at a high level across large diagnostic groups [15]. With mechanistic treatment trials on the horizon, their validation as potential disease-specific outcome measures now requires the implementation of quantitative oculomotor assessments in genetically stratified longitudinal natural history studies and eventually in parallel with clinical outcome assessments and other biomarker modalities.

In the companion paper, we reviewed the published spectrum of quantitative oculomotor assessments, and have provided consensus recommendations and technical guidelines for a core set of oculomotor paradigms and parameters for validation in future clinical studies, across ataxia genotypes (Garces et al. 2022a submitted). This study is a complementary review, focusing on studies reporting on quantitative oculomotor assessments for specific ataxia genotypes. It provides disease-specific recommendations for selecting the most promising quantitative oculomotor parameters as of yet, and integrates available cross-validations with other measures of disease severity to facilitate future validation in genotype-specific natural history or interventional studies. To facilitate this validation process, we systematically review the patterns of oculomotor and vestibular abnormalities across hereditary ataxias, and how they relate to other measures of disease severity (such as clinical scores, disease duration, and/or imaging). A particular focus for this work is on the responsiveness to disease progression and treatment effects.

## Material and Methods

### Data Sources and Searches

We searched MEDLINE (via PubMed) for articles using text words and controlled vocabulary terms related to research studies reporting on oculomotor and/or vestibular function in hereditary ataxia. A detailed description of the search strategy can be found in Appendix 1. Our search was updated through May 13, 2021.

### Study Selection and Quality Rating

**Fig. 1:**
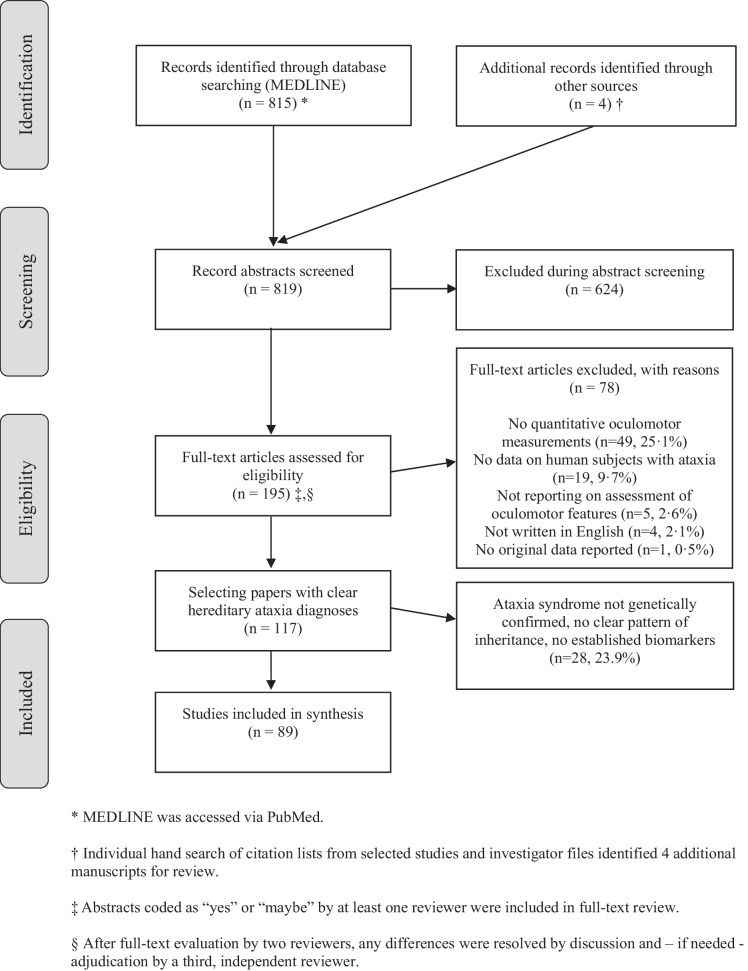
Citation search and selection flow diagram

Articles were selected by two independent raters (PG and AAT) using pre-determined inclusion criteria and a structured protocol (see Appendix 1). Our focus was on studies reporting on *quantitative* oculomotor and/or vestibular testing in *hereditary* ataxia. For this review, we included only studies with well-defined patient cohorts: (a) with genetically confirmed hereditary ataxia or (b) (if no genetic testing was available) ataxias with either a positive family history with a clear pattern of inheritance (autosomal dominant, autosomal recessive, X-linked recessive) or (c) with established and specific diagnostic biomarkers (as e.g., alpha-fetoprotein (AFP) in ataxia telangiectasia and ataxia with ocular motor apraxia 2 [16]), as illustrated in the PRISMA flow chart (Fig. 1). We calculated inter-rater agreement on full-text inclusion using Cohen’s kappa.

A quality rating of included studies was performed based on eight predefined quality criteria covering items related to (i) study cohort, (ii) data acquisition, and (iii) data analysis (see Appendix 2). An overall study quality rating (high, moderate, low) was derived from this quality assessment.

### Data Extraction, Data Synthesis, and Statistical Analysis

Information extracted from each eligible article included the type of study conducted, sample size and disease cohort studied, the oculomotor paradigms applied, and the recording device(s) used. We also searched for correlations between oculomotor parameters and anchor measures of disease severity such as clinical parameters (clinical scales, various questionnaires, disease duration, age of onset), biological determinants (e.g., CAG repeat length in polyQ diseases), and/or (MR) imaging markers. This study reports in accordance with PRISMA guidelines [17].

From each publication, we retrieved key information on the type of oculomotor paradigms performed and rated their potential responsiveness as outcome measures along four dimensions. Specifically, we assessed whether oculomotor parameters (i) discriminated significantly from healthy control groups, (ii) correlated to anchor measures of disease severity, (iii) captured progression over time (longitudinal observational studies), and/or (iv) captured modulation of progression by an intervention (clinical treatment trials). Correlations with anchor measures were classified for studies reporting Pearson correlation coefficients or Spearman correlation coefficients (strong: *r* ≥ 0.7; moderate: *r* ≥ 0.4 and *r* < 0.7; weak: *r* ≥ 0.1 and *r* < 0.4) [18]. For each oculomotor paradigm, a level of recommendation was graded according to the four criteria described above: Paradigms with high (or even disease-specific) discriminatory power and strong correlations with any measure of disease severity, or sensitivity to change (significant changes in observational or interventional studies) were recommended as “priority 1” for a given disease. Paradigms with low discriminatory power or—if any correlation analyses were performed—mild-to-moderate correlations with disease severity were considered “priority 2.” Paradigms without discriminatory power between patients and controls were “not recommended,” irrespective of correlations with disease severity measures. Correlations that were derived from pooled patient populations (i.e., merging several hereditary ataxias)—as reported in two publications [10, 19]—were not considered.

We focused on those five oculomotor domains that we have recommended for use in hereditary ataxia in the companion paper (Garces et al. 2022a, submitted), i.e., (i) pursuit eye movements (PEM), (ii) saccadic eye movements (SEM), (iii) fixation (including spontaneous nystagmus (SN) and saccadic intrusions (SI), (iv) eccentric gaze holding (looking for gaze-evoked nystagmus (GEN)), and (v) rotational vestibulo-ocular reflex (as measured with the quantitative head-impulse test (qHIT)).

## Results

### Overview of Studies

Our search identified 819 citations, of which 624 (76.2%) were excluded at the abstract level and 78 (9.5%) at the full-text manuscript level. From the 117 studies included after the full-text review, 89 studies (representing 10.9% of all manuscripts) reported on patients with either genetically confirmed hereditary ataxia or (if no genetic testing was available) ataxia with either a positive family history with a clear pattern of inheritance (autosomal dominant, autosomal recessive, X-linked recessive) or with established and specific biomarkers. Details on the study design and on specific disorders included are listed in Table 1. The overall study quality with respect to the predefined criteria (see Appendix 2) was judged “high” in 22 studies, “moderate” in 25 studies, and “low” in 42 studies (see Appendix 3, Table S1 for details).

**Table 1:** Overview of study design and clinical population across studies

	Studies (*n*)	Patients (*n*)
Gender		
Female	60	565
Male	59	614
Unclear	29	362
Total	89	1541
Study design—timeline		
Prospective	84	1503
Retrospective	4	29
Unclear	1	9
Study design—location		
Monocentric	83	1492
Multicentric	6	49
Study type		
Case series	20	195
Case–control studies	57	1095
Single-case reports	2	2
Observational studies	2	112
Randomized controlled treatment studies	2	71
Non-randomized treatment studies	6	66
Included disorders	Genetically confirmed	Based on positive family history/biochemical markers
Friedreich ataxia	102	76
SCA1	39	NA
SCA2	421	NA
SCA3	268	NA
SCA6	117	NA
ADCA others	46^§^	51
A-T	43	42
ATLD	2	NA
AOA1	12	NA
AOA2	15	NA
NPC	45	12
EA	10	NA
HSP	2	NA
RFC1-related ataxia	11	NA
ARCA	NA	7
Various	NA	1^#^

*ADCA* autosomal-dominant cerebellar ataxia, *AOA* ataxia with ocular motor apraxia, *ARCA* autosomal-recessive cerebellar ataxia, *A-T* ataxia telangiectasia, *ATLD* ataxia telangiectasia-like disease, *EA* episodic ataxia, *HSP* hereditary spastic paraparesis, *NA* not available, *NPC* Niemann-Pick disease type C, *RFC-1* replication factor complex subunit 1, *SCA* spinocerebellar ataxia

^§^This included SCA7 (*n* = 5), SCA8 (*n* = 8), SCA17 (*n* = 15), SCA31 (*n* = 12), SCA37 (*n* = 2), and other SCAs not specified (*n* = 56 [65–72])

^#^One patient with “non-identified” genetic ataxia [73]

The studies included reported on a broad range of oculomotor paradigms that were obtained with a variety of recording techniques (see Appendix 3, Tables S2 and S4 and also the companion manuscript (Garces et al. 2022a, submitted) for details).

### Diagnostic Discriminatory Power

Discriminatory power for each combination of oculomotor paradigm and ataxia diagnosis are summarized in Table 2, focusing on discrimination from healthy controls, which was the most common statistical comparison performed across studies. A succinct overview of the key oculomotor alterations for each genotype, what we refer to as their oculomotor “fingerprint,” is provided in Table 3.

**Table 2:** Overview of discriminatory power across studies for the five selected oculomotor paradigms

	Pursuit eye movements (PEMs)		Saccadic eye movements (SEMs)				Saccadic intrusions (SIs)			Spontaneous nystagmus (SN)			GEN	qHIT
	Gain	Velocity ↓ and sacc. intrusions	Velocity	Latency	Accuracy	MGS: sacc. latency/AS: prop. errors	SWJ (frequency)	OF (frequency)	Micro-opsoclonus (frequency)	Hor SN (presence)	Vertical SN (presence)	Torsional SN (presence)	GEN (presence)	qHIT gain
**FRDA **[10, 19, 26, 33, 34, 44–49, 74, 75]	↓/↓↓	82–100%	→ /↓	↑	↓	↑	+ +	+ +		∅	∅-DBN (15–45%)		15–100%	↓↓↓
**SCA1 **[10, 19, 70, 76–78]			↓–↓↓		↓		+	∅		∅	∅		60–100%	→ /↓
**SCA2 **[8, 10, 20, 23, 37–40, 70, 77, 79, 80]			↓↓↓	↑	→ /↓	↑	20–30%	∅		∅	∅		20–40%	→
**SCA3 **[9, 10, 19, 32, 70, 77, 81–87]	↓ Vert/hor	100%	→ /↓	→	↓		↑, 43–64%	∅	+	∅/ +	∅/ + (DBN)		66–100%	↓↓
**SCA6 **[41, 67, 70, 77, 78, 88–91]	↓↓↓	+ +	→ /↓		↓		80–100%				DBN (50–100%)		100%	↑/↓*
**SCA17 **[42]	↓		→		↓	↑							33%	
**A-T **[16, 50–52, 92, 93]	↓↓	+ +		↑	↓	↑	31–85%	50–69%		38%, PAN (62%)	DBN (43–46%)	100%	50%	
**NPC **[24, 43, 94, 95]	↓↓		↓↓↓ (vert), ↓ (hor)											→
**CANVAS/RFC-1 **[6, 12, 96–98]											DBN (45%)			↓↓/↓↓↓

Symbol legend: ∅ = finding absent; ( +)/ + / +  + / +  +  +  = finding present rarely (( +))/sometimes ( +)/in a significant fraction (+ +)/frequently (+ + +); ↑/↑↑/↑↑↑ = value increased mildly (↑)/moderately (↑↑)/strongly (↑↑↑); →  = value unchanged, ↓/↓↓/↓↓↓ = value decreased mildly (↓)/moderately (↓↓)/strongly (↓↓↓) compared to healthy controls

*amp* amplitude, *A-T* ataxia telangiectasia, *CANVAS* cerebellar ataxia-nystagmus-vestibular areflexia syndrome, *DBN* downbeat nystagmus, *FRDA* Friedreich ataxia, *GEN* gaze-evoked nystagmus, *hor* horizontal, *lat* latency, *NPC* Niemann-Pick disease type C, *OF* ocular flutter, *qHIT* quantitative head-impulse test, *PAN* periodic alternating nystagmus, *RBN* rebound nystagmus, *RFC-1* replication factor complex subunit 1, *sacc* saccadic, *SCA* spinocerebellar ataxia, *vert* vertical, *SN* spontaneous nystagmus, *SPV* slow-phase velocity, *SWJ* square-wave jerks

*Increased qHIT gain in mild cases, decreased qHIT gain in more severe cases

**Table 3:** Oculomotor “fingerprint” for selected hereditary ataxias

Disease	Domain	Key oculomotor changes
FRDA	PEM	• Moderately reduced velocity and gain
	Gaze holding	• Frequent saccadic intrusions (SWJ, OF) • Frequently impaired eccentric gaze holding (GEN)
	qHIT	• Strongly reduced qHIT gain
SCA1	SEM	• Moderately reduced velocity
	Gaze holding	• Frequently impaired eccentric gaze holding (GEN)
SCA2	SEM	• Strongly reduced velocity
	qHIT	• Preserved qHIT gain
SCA3	PEM	• Frequently reduced velocity and saccadic intrusions
	SEM	• Moderately reduced velocity of vertical saccades
	Gaze holding	• Saccadic intrusions in a significant fraction (SWJ, no OF) • Frequently impaired eccentric gaze holding (GEN)
	qHIT	• Moderately reduced qHIT gain
SCA6	PEM	• Frequent gain reductions
	Gaze holding	• Frequent saccadic intrusions (SWJ) and spontaneous nystagmus (DBN) • Frequently impaired eccentric gaze holding (GEN)
	qHIT	• Markedly reduced qHIT gain
A-T	PEM	• Moderately reduced gain, reduced velocity in a significant fraction
	Gaze holding	• Frequent saccadic intrusions (SWJ, OF) • Impaired straight-ahead fixation (SN) and eccentric gaze holding (GEN) in a significant fraction
NPC	PEM	• Moderately reduced gain
	SEM	• Strongly reduced velocity for vertical saccades
	qHIT	• Preserved qHIT gain
CANVAS/RFC1-related ataxia	qHIT	• Strongly reduced qHIT
	Gaze holding	• Impaired straight-ahead fixation (DBN) in a significant fraction

*A-T* ataxia telangiectasia, *CANVAS* cerebellar ataxia-nystagmus-vestibular areflexia syndrome, *DBN* downbeat nystagmus, *FRDA* Friedreich ataxia, *GEN* gaze-evoked nystagmus, *HSN* head-shaking nystagmus, *NPC* Niemann-Pick disease type C, *OF* ocular flutter, *qHIT* quantitative head-impulse test, *SCA* spinocerebellar ataxia, *SEM* saccadic eye movement, *SN* spontaneous nystagmus, *PEM* pursuit eye movement, *SWJ* square-wave jerks

### Correlation with Disease Severity Measures

Twenty-seven studies reported on at least one correlation with a measure of disease severity for specific hereditary ataxias, most frequently using SEM paradigms (*n* = 21 studies), followed by head-impulse testing (*n* = 6 studies) and PEM (*n* = 5 studies; see Table 4 for all paradigms). Disease severity measures were most often clinical scores (*n* = 18 studies; SARA in 9 studies, for more detailed information, see Table 4) or chronological measures (such as age, disease duration, or time to (calculated or observed) symptom onset, *n* = 17 studies), followed by genetic stratification (usually repeat expansion size, *n* = 9 studies) and imaging measures of atrophy (including MR imaging in 5 studies) as summarized in Table 4. The overall study quality was high (*n* = 8) or moderate (*n* = 12) in most cases, with low study quality that was noted in a minority of studies (*n* = 7) only.

**Table 4:** Overview of the number of studies reporting on correlation analyses between the five selected oculomotor parameters and other clinical information

	Studies (*n*)	Subjects (*n*)
Any correlation analysis performed		
Yes	27	671
No	62	870
Oculomotor parameters considered for correlation analyses		
Pursuit EM	5	187
Saccadic EM	21	636
Saccadic intrusions	3	89
Spontaneous nystagmus	1	73
Gaze-evoked nystagmus	2	129
Head-impulse test	6	126
Oher variables used in the correlations		
*Clinical scores*		
SARA	9	270
ICARS	4	114
FARS	4	33
A-T index	1	33
SLCLC	2	33
Others^§^	2	155
Any clinical score	18	459
*Patient-reported outcomes*		
VISATAX questionnaire	1	21
*Other clinical information*		
Disease duration	11	254
Age at symptom onset	7	194
Current age	7	189
Time to manifestation	3	164
Any other clinical parameter	17	519
*Imaging parameters*		
MRI-based	5	97
Optical coherence tomography (OCT)	1	31
*Laboratory parameters*		
Genetic testing (CAG-repeat length)	9	318

*A-T* ataxia telangiectasia, *EM* eye movement, *ICARS* International Cooperative Ataxia Rating Scale, *FARS* Friedreich Ataxia Rating Scale, *SARA* Scale for the Assessment and Rating of Ataxia, *SLCLC* Sloan Low-Contrast Letter Chart

^§^Other clinical scores included SCAFI/CCFS/NESSCA/INAScout [9] and a clinical ataxia score not further specified [23]

Overall, the majority of correlations identified were related to spinocerebellar ataxia (SCA) type 2 (SCA2), SCA3, or Friedreich ataxia (FRDA) (see Appendix 3, supplementary table S5 for all 105 significant correlations found in 20 studies), and the strength of correlations was high (*n* = 36) to moderate (*n* = 41) in most cases (see Table 5 and Appendix 3, supplementary table S6 for details). Disease severity measures were most frequently correlated with parameters of saccadic eye movements (including latency, peak velocity, accuracy, and the regression slope of saccadic peak duration vs. amplitude; 67/105 correlations (64%)), to parameters of pursuit eye movements (focusing on PEM gain in most cases, 11/105 correlations (10%)), to the gain obtained in quantitative head-impulse testing (qHIT; 8/105 correlations (8%)), and to the slow-phase velocity of gaze-evoked nystagmus (11/105 (10%)). Correlations taking fixation stabilities (SI or SN) into account were infrequently found (8/105 (8%)).

**Table 5:** Number of studies reporting significant correlations between oculomotor parameters and other clinical information for specific hereditary ataxias

	PEM	SEM	SI	SN	GEN	qHIT
Correlation with clinical scores						
SARA		3 (SCA3 (2 ×), SCA2) [9, 32, 37]			2 (SCA3 (2 ×)) [9, 32]	1 (SCA3) [9]
ICARS	2 (SCA3/SCA17) [9, 42]	2 (SCA3, SCA2) [9, 42]		1 (SCA3) [9]	1 (SCA3) [9]	2 (SCA3, SCA6) [9, 41]
FARS		4 (FRDA) [33–36]				
A-T index	1 (A-T) [52]					
SLCLC		2 (FRDA) [33, 35]	1 (FRDA) [34]			
Others^§^		2 (SCA3, SCA2) [9, 23]		1 (SCA3) [9]	1 (SCA3) [9]	1 (SCA3) [9]
Correlation with other clinical parameters						
Disease duration	2 (SCA3, SCA17) [32, 42]	4 (FRDA, SCA17, SCA2, SCA3) [23, 32, 33, 42]			1 (SCA3) [32]	
Age at symptom onset		2 (both SCA2) [23, 37]	1 (FRDA) [34]			
Current age	1 (A-T) [52]					
Time to manifestation		3 (SCA3, SCA2 (2 ×)) [9, 39, 40]			1 (SCA3) [9]	1 (SCA3) [9]
Correlation with imaging parameters						
MRI-based		3 (SCA2, NPC (2 ×)) [20–22]				
OCT		1 (NPC) [43]				
Correlation with laboratory parameters						
Genetic testing		6 (FRDA, SCA2 (4 ×), SCA3) [8, 23, 32, 33, 37, 39]				

A total of 20 studies were included in this table. The ataxia diagnosis investigated in each study is indicated in brackets. A summary of correlations identified for additional oculomotor paradigms is provided in Appendix 3, supplementary Table S3. Two studies reported on correlations after pooling genetically confirmed hereditary ataxia syndromes only, and thus were not considered here [10, 19]

*A-T* ataxia telangiectasia, *FARS* Friedreich Ataxia Rating Scale, *FRDA* Friedreich ataxia, *GEN* gaze-evoked nystagmus, *HSN* head-shaking nystagmus, *ICARS* International Cooperative Ataxia Rating Scale, *NPC* Niemann-Pick disease type C, *OCT* optical coherence tomography, *OKN* optokinetic nystagmus, *qHIT* quantitative head-impulse test, *rVOR* rotational VOR, *SARA* Scale for the Assessment and Rating of Ataxia, *SCA* spinocerebellar ataxia, *SEM* saccadic eye movements, *SI* saccadic intrusions, *SLCLC* Sloan Low-Contrast Letter Chart, *SN* spontaneous nystagmus, *PEM* pursuit eye movement, *SWJ* square-wave jerks, *VOR* vestibulo-ocular reflex, *VVOR* visually enhanced VOR, *VORS* vestibulo-ocular reflex suppression

^§^Other clinical scores included SCAFI/CCFS/NESSCA/INAScout [9] and a clinical ataxia score not further specified [23]

On MR imaging, SEM (peak velocity, gain) correlated with pontine volume/anterior–posterior pontine diameter in pre-symptomatic and symptomatic SCA2 [20] and with total cerebellar volume/gray matter [21] and various brainstem measurements (midbrain midsagittal area, pontine-midbrain ratio) in NPC [22]. For other oculomotor paradigms, no correlations with (MR) imaging were identified.

Importantly, for a number of combinations of genotypes and oculomotor paradigms, no data was available from the literature review, and therefore, no recommendations could be provided.

### Sensitivity to Disease Progression and Treatment Effects

In contrast to discriminatory or correlative cross-sectional studies, longitudinal studies monitoring disease progression (*n* = 2, both SCA2 [8, 23]) or treatment responses (*n* = 8, NPC, autosomal-dominant cerebellar ataxia (ADCA), episodic ataxia (EA) 4, A-T (2x), SCA2 (2x), FRDA) [24–31]) were rare. The two observational studies in SCA2 monitored horizontal visually guided saccades over variable/different timescales, demonstrating a significant decrease in saccade peak velocity and saccade accuracy over a period of 5 years [8], whereas saccade latency increased significantly over a 5-year period [8], but not over a shorter follow-up period of 1 year as reported in a different study [23].

Saccadic intrusions were reduced in peak velocity, frequency, and amplitude after memantine treatment in ADCA [28]; PEM and gaze holding were improved after application of gabapentin in EA4 [25]; vestibulo-ocular reflex time constant (VOR Tc) and SN slow-phase velocity was decreased after treatment with 4-aminopyridine in A-T patients [29], and saccade latencies were reduced after zinc sulphate supplementation in SCA2 [30]. In contrast, no positive effect of idebenone on saccadic intrusions was noted in FRDA [26], as well as for neurorehabilitation [27] or lisuride [31] on SEM in SCA2.

### Genotype-Specific Recommendations for Further Validation

To provide guidance in selecting suitable oculomotor paradigms for validation in genetically stratified natural history studies, we prioritized each paradigm per ataxia genotype, based on its discriminatory power, correlations with parameters of disease severity, and sensitivity to change (see “Material and Methods”). Overall, recommendations strongly depended on the specific oculomotor paradigm and disorder (Table 6). While SEM yielded generic oculomotor parameters across most ataxia types, recommendation of other paradigms was limited by the scarcity of cross-validating correlation analyses in most genotypes, even in more frequent ataxias such as SCA1, RFC1, or AOA1 and 2. SCA3 presents the most widely explored ataxia, with studies substantiating the recommendation of SEM, GEN, and qHIT [9, 32]. A broad range of parameters have also been assessed in both symptomatic and even pre-symptomatic SCA3 patients, with significant correlations for SEM, PEM, SN, gaze holding, and head-impulse testing [9]. Other recommended oculomotor paradigms were SEM [33–36] and SI for FRDA [34], SEM for SCA2 [8, 20, 23, 37–40], qHIT for SCA6 [41], SEM and PEM for SCA17 [42], and SEM for NPC [21, 22, 43]. Focusing on observational studies, faster progression rates of saccadic slowing were associated with higher trinucleotide CAG repeat expansions in SCA2 patients [8].

**Table 6:**
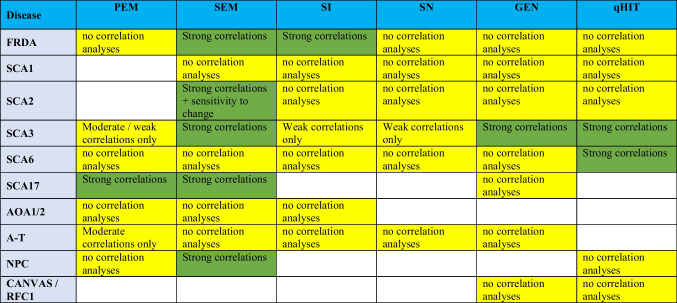
Genotype-specific recommendation of paradigms

Priority 1 requires data supporting high discriminatory power and at least one confirmed strong correlation, or sensitivity to change in observational or interventional studies. Priority 2 indicates that studies have found moderate to low discriminatory power or—if performed—the level of significance of correlation analyses being moderate or low only. Parameters that had no discriminatory power are not recommended. Priority 1 paradigms are marked in dark green, whereas priority 2 paradigms are marked in light yellow. Areas of uncertainty (i.e., paradigms that have not been quantitatively assessed in specific diseases) are shown in white

*AOA* ataxia ocular motor apraxia, *A-T* ataxia telangiectasia, *CANVAS* cerebellar ataxia-nystagmus-vestibular areflexia syndrome, *FRDA* Friedreich ataxia, *GEN* gaze-evoked nystagmus, *NPC* Niemann-Pick disease type C, *qHIT* quantitative head-impulse test, *SCA* spinocerebellar ataxia, *SEM* saccadic eye movement, *SN* spontaneous nystagmus, *PEM* pursuit eye movement, *RFC-1* replication factor complex subunit 1, *SI* saccadic intrusion

Noteworthy, in selected disorders, the fraction of patients that received a diagnosis based on either established biomarkers or a positive family history only was significant. Specifically, for FRDA (57%, 102/178 patients) and for A-T (51%, 43/85), only about half of patients had genetically confirmed hereditary ataxia, while in six studies that reported on FRDA patients [44–49] and in 3 studies on A-T patients [50–52], diagnosis was based on biomarkers/positive family history only. While this lack of genetically confirmed diagnosis increases the risk of misclassification due to overlapping phenotypes between distinct ataxias, all significant correlations reported for FRDA (see Table 5) were obtained from studies with genetically confirmed FRDA. For A-T, few correlations were reported (see Table 5), but originated from a study cohort that did not receive genetic testing [52].

## Discussion

Given that oculomotor and vestibular abnormalities are increasingly identified in hereditary ataxias [1], quantitative assessments have been of growing interest over the last few years. In this systematic review, we have examined the potential of quantitative oculomotor parameters as digital-motor outcome measures in specific hereditary ataxias, and made a range of suggestions for future validation studies based on gaps in our present knowledge. Specifically, we have shown that characteristic oculomotor abnormalities can be quantified by the use of eye movement recordings in hereditary ataxias, and that they are not only useful in differentiating between distinct underlying disorders, but can, in some cases, also capture disease severity and other clinical characteristics. Importantly, the pattern of oculomotor and vestibular alterations depends on the patient population studied.

### Disease-Specific Selection of Oculomotor Paradigms

Currently, our understanding of the value of a given oculomotor paradigm for specific hereditary ataxias with regards to its ability to discriminate ataxia patients from healthy controls, to capture progression over time or modulation of progression by an intervention and any correlations with other known measures of disease severity, is far from being complete. Historically, certain paradigms have been preferred based on hallmark signs identified by bedside testing as, e.g., very slow saccades in SCA2 [53] or saccadic intrusions in A-T [14]. This has significantly affected the selection of quantitative oculomotor parameters in previous studies. Whereas for certain disorders such as FRDA, SCA2, SCA3, SCA6, and A-T, we were able to retrieve descriptions of oculomotor abnormalities for a broad range of paradigms, in other disorders such as AOA1/2, NPC, or RFC1-related ataxia previous studies focused on only a limited number of paradigms. Table 6 may serve as a valuable starting point in guiding the choice of oculomotor paradigms for future validation in natural history studies, and eventually clinical trials, as it can help to prioritize across oculomotor paradigms in selecting those that are likely to be of most value, based on existing data. In designing future clinical trials, many aspects will affect the application of oculomotor testing, including competition with other domains to be assessed (e.g., appendicular motor or cognitive assessment), consideration of subject fatigue, stage of disease (e.g., assessments in non-ambulatory patients), and the infrastructure available at the various clinical sites. Furthermore, when selecting specific oculomotor paradigms, potential synergies with other measured information should be considered, as discussed in the next section.

### Various Oculomotor Assessments Are Correlated with Other (Clinical) Parameters

Construct validity of any outcome assessment requires a correlation with other measures of disease severity [54]. Such correlations were not homogeneously investigated across the spectrum of oculomotor paradigms, but were skewed towards a few paradigms and hereditary ataxia disorders. Specifically, significant correlations were most consistently and frequently observed in studies with SCA2, SCA3, and FRDA and were rated as moderate or strong in the majority of cases (73%).

The range of parameters that has been shown to correlate with eye movement assessments in the ataxias reported here was broad, including clinical scores rating disease severity, aspects of disease onset/duration, triplet size in repeat expansion disorders, and MRI-based biomarkers quantifying disease pathophysiology. More than half of all significant correlations were linked to saccadic eye movements and were identified in FRDA, NPC, SCA2, SCA3, and SCA17. Significant correlations using other oculomotor parameters such as PEM (noted in A-T and SCA3), qHIT (SCA3 and SCA6), GEN (SCA3), or saccadic intrusions (SCA3) were also reported, but were only investigated in a few studies and diseases. This emphasizes the gaps present in the current literature.

Existing data demonstrates significant correlations of quantitative oculomotor parameters with clinical scores such as SARA, ICARS, and FARS [9, 33, 36, 37, 41, 42]. Currently used clinical scores in hereditary ataxia do not take oculomotor abnormalities into account at all (as SARA [55] or FARS [56]) or represent only one out of several subscales, thus having limited impact on the overall score (as in ICARS [57]). Therefore, the correlations identified by our systematic review indicate that quantitative oculomotor parameters not only reflect damage to specific oculomotor systems, but also capture the severity of the underlying disease across multiple motor domains (as mainly reflected by SARA, FARS, and other clinical scores).

Likewise, changes in oculomotor parameters were also shown to relate to disease duration as a measure of disease severity in FRDA (SEM [33]), SCA2 (SEM [23]), SCA3 (PEM, SEM, and GEN [32]), and SCA17 (PEM and SEM [42]). Such correlations indicate potential responsiveness of the respective quantitative oculomotor parameter over time, and longitudinal studies are recommended to further validate sensitivity to change in trial-relevant time intervals. Importantly, various oculomotor parameters were altered in pre-symptomatic carriers in SCA2 (SEM [39, 40]) and SCA3 (SEM, GEN, and qHIT [9]), and even correlated significantly with the estimated or observed time to manifestation. This highlights the potential utility of using these parameters as outcome measures in early disease stages where disease-modifying therapies are probably most effective.

Correlations between MR imaging and oculomotor paradigms were investigated in SCA2, FRDA, and NPC only, while data is lacking for other hereditary ataxias. Noteworthy, significant correlations were identified only for SEM in SCA2 and NPC, but not in FRDA [49]. Specifically, various SEM parameters correlated with brainstem alterations (i.e., pontine volume and the anterior–posterior pontine diameter) in pre-symptomatic and symptomatic SCA 2 [20] and with both cerebellar (total cerebellar volume/gray matter [21]) and various brainstem measurements (midbrain midsagittal area, pontine-midbrain ratio) [22] in NPC. Furthermore, changes in thickness of different retinal layers on optical coherence tomography (OCT) correlated with SEM in NPC [43]. Thus, in selected hereditary ataxias, a link between structural changes on brainstem and cerebellar MR imaging and eye movement properties could be achieved and thus may be used as a parameter to reflect more global disease damage in future studies.

### Quantitative Oculomotor Parameters Suitable to Monitor Disease Progression and Treatment Response

Parameters suitable for monitoring disease progression or improvement (i.e., biomarkers) must be robust and sensitive to change. Noteworthy, the SARA has been shown to reflect progression in hereditary ataxias such as SCA1 [58–60], SCA2 [58, 60], SCA3 [58, 60], SCA6 [60], FRDA [61], and even in pre-symptomatic SCA1 carriers [62]. While validation of quantitative oculomotor parameters was often limited to cross-sectional analysis, only a small number of studies have directly assessed these properties by investigating oculomotor parameters in longitudinal studies [8, 23].

We identified two studies reporting on the suitability of oculomotor parameters for monitoring disease progression in SCA2. Specifically, significant decreases in saccade peak velocity and saccade accuracy and increase in saccade latency were detected over a period of 5 years [8] but was not significant over a shorter—more trial-relevant—follow-up period of 1 year [23] (despite an average decrease in peak velocity of 8°/s per year, *n* = 30 SCA2 patients). In SCA3, two studies compared oculomotor parameters in patients at different disease stages (e.g., pre-symptomatic far from onset, pre-symptomatic close to onset and symptomatic) cross-sectionally. Specifically, in (pre)symptomatic SCA3 carriers, vertical PEM gains, vertical SEM slopes, horizontal SN and GEN slow-phase velocity, and horizontal video-HIT gains showed stronger alterations in those patients who were symptomatic or became symptomatic soon after oculomotor testing [9, 32].

Given this scarcity of longitudinal data, longitudinal validation studies are urgently needed to improve our knowledge of the sensitivity to disease progression of selected oculomotor paradigms and parameters. For a given paradigm and genotype, these studies may have to be tailored to specific disease stages, given that some oculomotor abnormalities are present through almost the entire disease (e.g., saccadic slowing in SCA2), while others rarely occur neither in the pre-symptomatic stage nor in severe disease stages (e.g., saccadic intrusions in SCA2) [63]. Importantly, the large effort of such longitudinal validation studies should be undertaken with the clear aim of regulatory acceptance, focusing on trial-relevant assessment intervals, and complementing quantitative oculomotor assessments by necessary clinician-, patient-, and possibly performance-related outcomes ([64]). In a separate publication (see companion paper Garces et al. 2022a submitted), we provided guidelines for the measurement of those quantitative oculomotor parameters we have reviewed here, including recommendations on acquisition paradigms, measurement conditions, parameter extraction, and biases to consider.

## Conclusions

To our knowledge, this is the first systematic literature review on the role of quantitative oculomotor parameters in hereditary ataxias. Where available, we have summarized information on case–control discrimination correlations with measures of disease severity, and evidence of longitudinal and treatment studies separately for each disease and paradigm. Based on this, we provide disease-specific recommendations for selecting the most promising oculomotor paradigms for preparation of future clinical trials, which are firmly placed on the horizon. Additionally, this review highlights current gaps in our knowledge and the critical need for well-designed, genotypically stratified longitudinal validation studies aiming for regulatory approval.

### Supplementary Information

Below is the link to the electronic supplementary material.

Supplementary file1 (DOCX 502 KB)

## Data Availability

The data that support the findings of this study are available from the corresponding author upon reasonable request.
