# Supplementary material for: Quantitative Oculomotor Assessment in Hereditary Ataxia: Discriminatory Power, Correlation with Severity Measures, and Recommended Parameters for Specific Genotypes
Source: Cerebellum. 2023 Jan 14;23(1):121–35. doi: 10.1007/s12311-023-01514-8 (PMC10864420; doi:10.1007/s12311-023-01514-8)
Supplement: Supplementary file 1 — Supplementary file1 (DOCX 502 KB) [file 12311_2023_1514_MOESM1_ESM.docx]

## Online-Only Supplemental Materials

## Appendix 1: Electronic search strategy, coding-scheme for the systematic review and data analysis

**The search strategy was designed by a clinical investigator with relevant domain expertise in neurology (AAT) and has been previously described in the companion paper (Garces et al. 2022, in preparation).**

**We searched MEDLINE for English-language articles, using the following strategies with the following components: (1) defining the clinical syndrome (i.e., ataxia), (2)** ocular motor or vestibular features**, and (3) quantitative assessments. We did not expressly search for hereditary ataxia syndromes,** as this was found to result in omitting most relevant studies because they did not refer to the genetic background in a uniform fashion**. We also performed a manual search of reference lists from eligible articles, and contacted corresponding authors where necessary. We did not seek to identify research abstracts from meeting proceedings or unpublished studies.**

MEDLINE Search *(accessed via PubMed at www.ncbi.nlm.nih.gov/pubmed)*

(ataxia OR ataxic OR (gait AND impairment)) AND ((eye movements) OR (ocular motor) OR oculomotor OR vestibular OR saccade OR (smooth pursuit) OR (vestibulo-ocular reflex) OR VOR OR optokinetic OR nystagmus OR gaze OR head impulse OR caloric) AND (quantitative OR recording OR recordings OR quantified OR measured)

Search Results

Our search identified 819 unique citations, of which 624 (76.2%) were excluded at the abstract level (Figure 1, main manuscript). A record was excluded only if two scorers (PG, AAT) recommended exclusion (detailed list of predefined reasons for exclusion shown below). We did not demand concordance on reason for abstract exclusion, but, among those abstracts with concordant reasons for exclusion (63.3%, n=395), the distribution was as follows: 44.2% had no data on human subjects with ataxia**; 6.6% were not reporting on the assessment of oculomotor /vestibular features; 6.1% were not reporting on quantitative measurements; 5.8% had no original data and 0.6% were not in English.**

We sought to examine 195 full manuscripts (this included 4 articles identified by hand-search). After initial screening, there were a total of 10 disagreements about study inclusion for the two reviewers (PG and AAT, kappa=0.89 [95% CI: 0.85 – 0.94]). These differences were resolved by discussion. Overall agreement on reason for exclusion was 93.1%. In order to achieve concordance on reason for full-text exclusion, we resolved differences by discussion.

At the end of our full-text review, 702 were excluded and 117 were considered eligible (Figure 1, main manuscript). These eligible studies represented 14.3% of the total (n=819). Among all full-text manuscripts excluded (40.0%), the distribution of reason for exclusion was as follows: 25.1**% were not reporting on quantitative measurements;** 9.7% had no data on human subjects with ataxia**; 2.6% were not reporting on the assessment of oculomotor /vestibular features; 2.1% were not in English and 0.5% had no original data.**

**From those 117 manuscripts, 89 (76.1%) reported** on patients with either genetically-confirmed hereditary ataxia or (if no genetic testing was available) ataxia with either a positive family history with a clear pattern of inheritance (autosomal dominant [AD], autosomal recessive [AR], X-linked recessive [XR]) or with established biomarkers. Only those studies were selected for further analyses.

### Coding schema for abstract and full-text reviews

**All gathered literature was subject to title/abstract screening by two independent reviewers (PG and AAT). Full-text screening was then applied to all citations considered eligible or possibly eligible by at least one reviewer. Two independent reviewers (PG and AAT) determined whether full-text manuscripts were eligible and, if not, provided a reason for exclusion. Differences were resolved by discussion and consensus. AAT completed a hand search of the reference lists of selected articles for additional citations. For citations identified by hand search, the full process was repeated iteratively until no additional manuscripts were found for inclusion. We calculated inter-rater agreement on full-text inclusion using Cohen’s kappa [1]. A formal review protocol was not registered or posted.**

Abstract Review Coding Rules

1) Coding status options are “Yes”, “No”, “Maybe”. We will review full text of “Yes” and “Maybe”. The purpose of “Yes” vs. “Maybe” is to look at kappa values agreement on “Yes” vs. “Maybe”.

2) Err on the side of “Maybe” if there is doubt about a “No”; this is more conservative.

3) If there is only a title, exclude it only if you feel confident; otherwise code it as “Maybe”.

4) Each "No" or "Maybe" should be coded with a reason for exclusion.

5) Reasons for exclusion are listed below 0-5. Go through them in order from 0 to 5 for each abstract, coding the first reason for exclusion only, not multiple reasons for exclusion. Only code "0" for “not English” if you are sure it is “not English”.

6) Two independent raters will code reason for exclusion, but we will not mandate agreement on exclusion reason at the abstract level.

7) Occasionally an abstract seems inappropriate for another reason. In such cases, code as “other”. There should be few “other” codings.

Abstract Reasons for Exclusion

| 0 | not English | manuscript is not in English |
| --- | --- | --- |
| 1 | no data | review paper; no original patient data |
| 2 | not ataxia | No data on human subjects with ataxia |
| 3 | not oculomotor / vestibular | not reporting on the assessment of oculomotor and/or vestibular features |
| 4 | not quantitative | not reporting on quantitative oculomotor and/or vestibular measurements |
| 5 | other | any other reason abstract is not included |

Full-Text Review Coding Rules

1) Coding status options are “Yes” or “No”.

2) Each "No" should be coded with a reason for exclusion.

3) Reasons for exclusion are listed below 0-4. Go through them in order from 0 to 4 for each full text, coding the first reason for exclusion only, not multiple reasons for exclusion.

4) Two independent raters will code reason for exclusion, and we will mandate agreement on exclusion reason at the full text level.

5) Coding differences will be adjudicated or consensus will be developed through dialogue.

Full-Text Reasons for Exclusion

| 0 | not English | manuscript is not in English |
| --- | --- | --- |
| 1 | no data | review paper; no original patient data |
| 2 | not ataxia | No data on human subjects with ataxia |
| 3 | not oculomotor / vestibular | not reporting on the assessment of oculomotor and/or vestibular features |
| 4 | not quantitative | not reporting on quantitative oculomotor and/or vestibular measurements |

## Appendix 2: quality assessment for studies reporting on oculomotor findings in ataxia

Two independent reviewers (PG and AAT) rated all included studies with regards to their quality in reporting oculomotor testing in ataxia patients. Discrepancies were resolved by discussion. Based on the eight items listed below an overall quality rating (high, moderate, low) was assigned. “High quality” was defined as having high quality on items 1, 3-7 and a low risk-of-bias for item 8. Item 2 was not considered since, depending on the study design a control group may not be required (e.g. for treatment response studies). “Moderate quality” studies were defined as studies having at least moderate quality ratings for all three items (with „low“ or „high“ risk of bias for item 8). All studies that received a low quality rating in one or several items (items 1-7) or had an unknown risk of bias for item 8 were considered “low quality” studies.

**Criteria for the quality assessment of reporting oculomotor findings in ataxia**

**Study cohort related items**

1. Patient selection

high = underlying disorder in all patients included was confirmed by genetic testing

moderate = no genetic testing available but either positive family history with a clear pattern of inheritance (AD, AR, XR) or established and specific diagnostic biomarkers used

low = clinically- or imaging-based diagnosis, no genetic testing, no positive family history

2. Control group selection

high = control group age-matched, independent from test group, clinical assessment (exclusion of abnormal findings) provided in detail, patients serve as their own controls in longitudinal treatment trials or treatment and placebo groups were age-matched in placebo-controlled treatment trials.

moderate = age-matched, reportedly healthy but no details reported how this was assessed

low = one or several of the following: controls are not age-matched; no information about their age is available; no information about selection process is provided; controls are genetically undetermined relatives of the test patients included.

not available = no control group is provided

**Items related to data acquisition**

3. Rigidity of recording protocol applied

high = pre-specified recording protocol available for all parameters studied, identical protocol applied in all participants and sessions

moderate = pre-specified recording protocol available only for selected parameters or different pre-specified recording protocols applied in included patients and sessions.

low = no pre-specified recording protocol used or no information provided about the use of a recording protocol.

4. Description of recording parameters used

high = all recording parameters are reported with sufficient detail to reproduce the study

moderate = recording parameters are reported partially only

low = no information provided about recording parameters

5. Appropriateness of recording devices used for oculomotor testing

high = high temporal and spatial resolution, high signal-to-noise recording technique used (e.g. search coils, high-quality video-oculography with sampling rate of >=100Hz)

moderate = recording technique with moderate to high noise levels (e.g. electro-oculography, electro-nystagmography, low to middle performance video-oculography (sampling rate <100Hz)) or behavioral paradigm used (e.g. Subjective Visual Vertical)

low = no details about recording device reported or high noise levels described

**Items related to data analysis**

6. Description of data analysis

high = data analysis is described with sufficient detail to allow reproduction of study

moderate = data analysis description is provided, but details are lacking and thus is insufficient to allow reproduction of study

low = no information provided about how the data analysis was performed or inadequate analysis pipeline

7. Assessment of obtained test results for significance

high = statistical analysis using normative values obtained from a control group or baseline values (for longitudinal studies)

moderate = statistical analysis using normative values from published literature or from the manufacturer of the device used

low = no normative values available and/or no statistical analysis performed

8. Risk of bias for assessing test results

low = index test results interpreted in a blinded fashion (i.e., without knowledge if the underlying disorder was present or absent) or automated data analysis without rater’s input

high = index test results interpreted in a non-blinded fashion only and / or based on the rater’s subjective assessment (e.g. rating of overall pattern of qHIT traces).

unclear = no information provided about blinding of reviewers of test results

# Appendix 3 – additional tables

Supplementary Table 1 – quality assessment for studies reporting on oculomotor findings in ataxia – overview on all selected studies

| **Table S1: quality assessment for studies reporting on oculomotor findings in ataxia – overview on all selected studies** | | | | | | | | | |
| --- | --- | --- | --- | --- | --- | --- | --- | --- | --- |
| **Study** | **Patient selection** | **Control group selection** | **Rigidity of recording protocol applied** | **Description of recording parameters used** | **Appropriateness of recording devices used for OM /vestibular testing** | **Appropriateness of data analysis applied** | **Assessment of obtained test results for significance** | **Risk of bias for assessing test results** | **OVERALL study quality rating** |
| Alexandre et al. 2013 [2] | high | moderate | high | high | high | moderate | high | low | moderate |
| Anastasopoulos et al. 1998 [3] | low | moderate | high | high | high | high | high | high | low |
| Anderson et al. 2002a [4] | moderate | moderate | high | high | high | moderate | high | low | moderate |
| Anderson et al. 2002b [5] | high | low | high | high | moderate | high | high | low | low |
| Baloh et al. 1975 [6] | low | low | high | low | moderate | moderate | low | high | low |
| Baloh et al. 1978 [7] | low | low | high | low | moderate | low | low | unclear | low |
| Bargagli et al. 2021 [8] | high | moderate | high | high | high | high | high | low | high |
| Bour et al. 2008 [9] | high | moderate | high | high | high | high | high | low | high |
| Bremova et al. 2016 [10] | moderate | moderate | high | high | high | high | high | low | moderate |
| Bremova et al. 2015 [11] | moderate | high | high | moderate | high | low | high | low | moderate |
| Bürk et al. 1996 [12] | high | moderate | high | moderate | moderate | moderate | high | unclear | low |
| Bürk et al. 1997 [13] | moderate | moderate | high | moderate | moderate | moderate | high | unclear | low |
| Büttner et al. 1998 [14] | high | not applicable | high | high | moderate | high | low | high | moderate |
| Caspi et al. 2013 [15] | high | moderate | high | high | high | high | high | unclear | low |
| Chang et al. 2020 [16] | low | low | high | high | moderate | high | high | low | low |
| Choi et al. 2015 [17] | high | not applicable | high | moderate | moderate | low | low | unclear | low |
| Christova et al. 2008 [18] | high | moderate | high | high | high | moderate | high | low | moderate |
| Ciuffreda et al. 1985 [19] | low | not applicable | low | moderate | moderate | low | low | high | low |
| Clausi et al. 2013 [20] | high | moderate | high | high | high | moderate | high | unclear | low |
| Coin and Vance 2021 [21] | high | high | low | low | moderate | low | low | high | low |
| Costales et al. 2021 [22] | high | not applicable | high | moderate | high | moderate | low | low | low |
| Crane et al. 2000 [23] | low | low | high | high | high | high | high | low | low |
| Crowdy et al. 2000 [24] | low | high | high | high | high | high | high | low | low |
| Dakin et al. 2018 [25] | high | moderate | high | high |  | high | high | low | high |
| Dale et al. 1978 [26] | moderate | not applicable | high | moderate | moderate | moderate | low | high | moderate |
| de Oliveira et al. 2021 [27] | high | low | high | moderate | high | high | high | low | low |
| Ell et al. 1984 [28] | moderate | not applicable | high | moderate | moderate | moderate | low | high | moderate |
| Fahey et al. 2008 [29] | high | low | high | high | high | high | high | low | low |
| Federighi et al. 2011 [30] | high | high | high | high | high | high | high | low | high |
| Federighi et al. 2017 [31] | high | moderate | high | high | high | high | high | low | high |
| Fielding et al. 2010 [32] | high | moderate | high | high | high | high | high | low | high |
| Furman et al. 1983 [33] | low | low | high | high | moderate | low | high | unclear | low |
| Geisinger et al. 2021 [34] | high | high | high | high | high | high | high | low | high |
| Ghasia et al. 2016 [35] | high | low | high | moderate | high | high | moderate | high | low |
| Gomez et al. 1997 [36] | high | not applicable | high | high | high | moderate | low | high | low |
| Gordon et al. 2014 [37] | high | moderate | high | high | high | high | high | low | high |
| Havla et al. 2020 [38] | high | moderate | high | high | high | high | high | low | high |
| Hocking et al. 2014 [39] | high | moderate | high | high | high | high | high | low | high |
| Hocking et al. 2010 [40] | high | moderate | high | high | high | high | high | low | high |
| Hübner et al. 2007 [41] | high | moderate | high | moderate | high | high | high | low | moderate |
| Huh et al. 2015 [42] | high | moderate | high | high | high | high | high | low | high |
| Joiner et al. 2005 [43] | high | low | high | high | low | moderate | low | low | low |
| Kerber et al. 2005 [44] | high | not applicable | high | moderate | moderate | moderate | moderate | high | moderate |
| Kim et al. 2013 [45] | high | moderate | high | high | high | high | high | high | moderate |
| King et al. 2011 [46] | low | low | high | high | high | moderate | high | low | low |
| Lasker et al. 2005 [47] | moderate | moderate | high | high | high | moderate | high | low | moderate |
| Lemos et al. 2018 [48] | high | not applicable | high | high | high | low | low | low | low |
| Lewis and Crawford 2002 [49] | moderate | not applicable | high | moderate | high | high | low | low | low |
| Lewis et al. 1999 [50] | moderate | low | high | moderate | moderate | high | moderate | low | low |
| Lopez et al. 2019 [51] | low | moderate | low | moderate | moderate | moderate | high | low | low |
| Luis et al. 2016 [52] | high | high | high | high | high | high | high | low | high |
| Mariani et al. 2017 [53] | high | moderate | high | high | high | high | high | low | high |
| Matsuda et al. 2014 [54] | moderate | moderate | high | high | high | high | high | low | high |
| Matsuda et al. 2015 [55] | high | moderate | high | moderate | high | high | high | low | moderate |
| Moschner et al. 1994 [56] | low | moderate | high | high | moderate | moderate | high | low | low |
| Oh et al. 2001 [57] | high | low | high | high | moderate | moderate | moderate | low | low |
| Ohyagi et al. 2000 [58] | high | low | high | moderate |  | high | low | low | low |
| Pretegiani et al. 2018 [59] | high | moderate | high | high | high | high | high | low | high |
| Reetz et al. 2018 [60] | high | moderate | high | high | moderate | high | high | low | moderate |
| Ribai et al. 2007 [61] | high | high | high | high | moderate | moderate | high | unclear | low |
| Ribeiro et al. 2015 [62] | high | moderate | high | high |  | high | high | low | high |
| Rodríguez-Díaz et al. 2018 [63] | high | high | high | high | moderate | high | high | low | moderate |
| Rodríguez-Labrada et al. 2017 [64] | high | moderate | high | high | moderate | moderate | high | low | moderate |
| Rodríguez-Labrada et al. 2016 [65] | high | high | high | high | moderate | high | high | low | moderate |
| Rosini et al. 2013 [66] | low | high | high | high | high | high | high | low | high |
| Rufa and Federighi 2011 [67] | high | moderate | high | high | high | high | high | low | high |
| Sağlam and Lehnen 2014 [68] | low | low | high | high | high | high | high | low | low |
| Seifried et al. 2005 [69] | high | moderate | high | high | moderate | high | high | low | moderate |
| Serrano-Munuera et al. 2013 [70] | high | low | high | high |  | high | moderate | low | low |
| Shaikh et al. 2013 [71] | moderate | low | moderate | high | moderate | high | high | low | low |
| Shaikh et al. 2011 [72] | moderate | not applicable | moderate | low | moderate | moderate | low | unclear | low |
| Shaikh et al. 2009 [73] | high | low | high | high | high | high | high | low | low |
| Solomon et al. 2005 [74] | moderate | not applicable | high | moderate | high | low | low | unclear | low |
| Spieker et al. 1995 [75] | moderate | high | high | high | moderate | high | high | low | moderate |
| Takegoshi and Murofushi 2000 [76] | high | low | high | high | moderate | high | high | low | low |
| Takeichi et al. 2000 [77] | high | moderate | high | high |  | high | high | low | high |
| Terao et al. 2016 [78] | low | moderate | high | high | moderate | high | high | low | low |
| Terao et al. 2017 [79] | low | moderate | high | high | moderate | high | high | low | low |
| Velázquez-Pérez et al. 2011 [80] | high | high | high | high | moderate | high | high | low | moderate |
| Velázquez-Pérez et al. 2012 [81] | high | high | high | high | moderate | high | high | low | moderate |
| Velázquez-Pérez et al. 2014 [82] | high | moderate | high | high | moderate | high | high | low | moderate |
| Velázquez-Pérez et al. 2009 [83] | high | high | high | high | moderate | high | high | low | moderate |
| Walterfang et al. 2013 [84] | moderate | moderate | high | high | high | high | high | low | moderate |
| Walterfang et al. 2012 [85] | moderate | moderate | high | high | high | high | high | low | moderate |
| Wessel et al. 1998 [86] | low | moderate | high | high | moderate | high | high | low | low |
| Wiest et al. 2001 [87] | high | moderate | high | high | high | high | high | low | high |
| Wu et al. 2017 [88] | high | high | high | high | high | high | high | high | high |
| Yue et al. 1997 [89] | high | low | high | high | moderate | low | low | low | low |
| Zee et al. 1976 [90] | moderate | low | high | high | high | low | low | high | low |

Supplementary Table 2 – Meta-data of included studies listed in alphabetical order

| **Table S2: meta-data of selected studies** | | | | | | | | |
| --- | --- | --- | --- | --- | --- | --- | --- | --- |
| **Author, year (citation)** | **Study population** | **Study location** | **Data collection (analysis)** | **Subjects (% females)** | **Mean age (SD)** | **Paradigms recorded** | **Recording device used** | **Special comments** |
| Alexandre et al. 2013 [2] | FRDA, SCA1, SCA3 | monocentric | Prospective, case-control-study | 21 (43%) | 48 (14) | VGS, SI, | VOG (mobile EBT) |  |
| Anastasopoulos et al. 1998 [3] | mixed hereditary (SCA1, other ADCA) and non-hereditary | monocentric | Prospective, case-control-study | 10 (NR) | 39 (10) | PEM, OVAR, | Search coils |  |
| Anderson et al. 2002a [4] | SCA2, EA2 | monocentric | Prospective, case-series | 4 (50%) | 54 (13) | PEM, VGS, | Search coils |  |
| Anderson et al. 2002b [5] | SCA8 | monocentric | Prospective, case-control-study | 3 (NR) | NR (NR) | rVOR, vVOR Tc | EOG |  |
| Baloh et al. 1975 [6] | FRDA, CA | monocentric | Retrospective, case-control-study | 15 (NR) | NR (NR) | PEM, VGS, SI, OKN, rVOR, SN, PN, GEN, RBN, CI | EOG |  |
| Baloh et al. 1978 [7] | AT | monocentric | Prospective, case-series | 6 (50%) | 11 (7) | PEM, VGS, OKN, rVOR, VORs, SN, CI | EOG |  |
| Bargagli et al. 2021 [8] | AOA2 | Monocentric | Prospective, case-control-study | 2 (50%) | 11 (1) | VGS, AS, GEN, RBN | VOG (ASL 504 eye tracker) |  |
| Bour et al. 2008 [9] | SCA6, other ADCA | monocentric | Prospective, case-control-study | 11 (45%) | 42 (15) | PEM, VGS, SI, SN, HN, GEN, | Search coils |  |
| Bremova et al. 2016 [10] | NPC | monocentric | Prospective, case-control-study | 8 (NR) | 27 (10) | qHIT, oVEMPs, cVEMPs, CI, SVV | VOG (EyeSeeCam, Interacoustics) |  |
| Bremova et al. 2015 [11] | NPC | monocentric | Prospective, non-randomized treatment study | 12 (42%) | 23 (5) | PEM, VGS, OKN, rVOR, | VOG (EyeSeeCam, Interacoustics) |  |
| Bürk et al. 1996 [12] | SCA1, SCA2, SCA3 | monocentric | Prospective, case-control-study | 24 (NR) | SCA1=34 (4) SCA2=35 (5) SCA3=41 (4) | VGS | EOG |  |
| Bürk et al. 1997 [13] | SCA1, SCA2, SCA3, other ADCA, LOCA | monocentric | Prospective, case-control-study | 48 (NR) | 44-58 (6-16) | VGS | EOG |  |
| Büttner et al. 1998 [14] | SCA1, SCA2, SCA3, SCA6 | monocentric | Prospective, case-series | 20 (NR) | 46 (12) | PEM, VGS, SI, OKN, rVOR, VVOR, VORs, SN, GEN, RBN, | EOG |  |
| Caspi et al. 2013 [15] | SCA3 | monocentric | Prospective, case-control-study | 10 (70%) | 49 (15) | VGS | Search coils |  |
| Chang et al. 2020 [16] | FRDA, SCA1, SCA2, SCA3, SCA6, other ADCA (not specified), AT, HSP, ARCA, MSA-C | monocentric | Prospective, case-control-study | 59 (54%) | 53 (19) | PEM | VOG (smartphone) |  |
| Choi et al. 2015 [17] | EA2 | monocentric | Prospective, case-series | 4 (50%) | 26 (12) | SN | VOG (SMI) |  |
| Christova et al. 2008 [18] | SCA6 | monocentric | Prospective, case-control-study | 9 (55%) | 43 (12) | PEM, VGS, SI, | Search coils |  |
| Ciuffreda et al. 1985 [19] | FRDA | monocentric | Retrospective, single case report | 1 (100%) | 23 | PEM, VGS, SI | EOG |  |
| Clausi et al. 2013 [20] | AOA2 | monocentric | Prospective, case-control-study | 2 (50%) | 39 (1) | VGS, SI, | VOG (NR) |  |
| Coin and Vance 2021 [21] | EA4 | monocentric | Prospective, non-randomized treatment study | 3 (67%) | 52 (14) | PEM, GEN | VOG (consumer digital camera) |  |
| Costales et al. 2021 [22] | RFC1-related ataxia | Monocentric | Retrospective, case series | 11 (64%) | 69 (4) | SN, qHIT | VOG (ICS impulse goggles, Natus) |  |
| Crane et al. 2000 [23] | FRDA, SCA3, SCA6, ARCA, EOCA | monocentric | Prospective, case-control-study | 11 (NR) | 48 (16) | rVOR, tVOR | Search coils |  |
| Crowdy et al. 2000 [24] | SCA1, SCA7, other ADCA, EOCA, LOCA | monocentric | Prospective, case-control-study | 8 (25%) | 43 (9) | VGS | VOG (NR) |  |
| Dakin et al. 2018 [25] | SCA6 | monocentric | Prospective, case-control-study | 15 (47%) | 67 (10) | SVV | NA |  |
| Dale et al. 1978 [26] | FRDA | Monocentric | Retrospective, case series | 2 (50%) | 19 (2) | PEM, SI, OKN, GEN, | EOG |  |
| de Oliveira et al. 2021 [27] | SCA3 (symptomatic/pre-symptomatic) | monocentric | Prospective, case-control-study | 73 (52%) | Symp=42 (9) presymp <4y= 33 (9)  Presymp >4y=27 (6) | PEM, VGS, SI, GEN, qHIT | VOG (eyeseecam, Interacoustics) | 38 pre-symptomatic carriers included (est. onset <4 years or >4 years) |
| Ell et al. 1984 [28] | FRDA | Monocentric | Prospective, case series | 10 (40%) | 25 (9) | PEM, VGS, SI, OKN, VORs, CI, | EOG |  |
| Fahey et al. 2008 [29] | FRDA | monocentric | Prospective, case-control-study | 20 (NR) | 35 (13) | PEM, VGS, SI, SN, GEN, qHIT | VOG (Micromedical Technologies) or search coils |  |
| Federighi et al. 2011 [30] | SCA2, LOCA | monocentric | Prospective, case-control-study | 15 (40%) | SCA2=44 LOCA=51 | VGS, AS, | VOG (ASL 504 eye tracker) |  |
| Federighi et al. 2017 [31] | ATLD | monocentric | Prospective, case-control-study | 2 (50%) | 45 (1) | VGS, SI, GEN, RBN | VOG (ASL 504 eye tracker) |  |
| Fielding et al. 2010 [32] | FRDA | monocentric | Prospective, case-control-study | 13 (NR) | 36 (9) | VGS, MGS, AS | Search coils |  |
| Furman et al. 1983 [33] | FRDA | monocentric | Prospective, case-control-study | 24 (54%) | NR | PEM, VGS, SI, OKN, rVOR, VVOR, VORs, GEN, RBN | EOG |  |
| Geisinger et al. 2021 [34] | SCA3 | Monocentric | Prospective, case-control-study | 21 (71%) | 59 (16) | qHIT, cVEMPs | VOG (ICS impulse goggles, Natus) |  |
| Ghasia et al. 2016 [35] | SCA3 | Monocentric | Prospective, case series | 12 (67%) | 50 (17) | PEM, VGS, SI, GEN | VOR (eyelink 1000 or Jazz Novo) |  |
| Gomez et al. 1997 [36] | SCA6 | Monocentric | Prospective, case series | 13 (NR) | NR | PEM, VGS, SI, rVOR, rVOR decay Tc, VORs, SN, GEN, | Search coils |  |
| Gordon et al. 2014 [37] | SCA3 | monocentric | Prospective, case-control-study | 10 (70%) | 49 (15) | qHIT | Search coils | Same patients as in Caspi et al. 2013 [15] |
| Havla et al. 2020 [38] | NPC | monocentric | Prospective, case-control-study | 31 (48%) | NPC1-P=24 NPC1-MC=50 | PEM, VGS, | VOG (eyeseecam, Interacoustics) | Including 17 asymptomatic mutation carriers |
| Hocking et al. 2014 [39] | FRDA | monocentric | Prospective, case-control-study | 13 (NR) | 36 (9) | VGS | Search coils | Same patients as in Fielding et al. 2010 [32] |
| Hocking et al. 2010 [40] | FRDA | monocentric | Prospective, case-control-study | 13 (NR) | 36 (9) | VGS | Search coils | Same patients as in Fielding et al. 2010 [32] |
| Hübner et al. 2007 [41] | SCA17 | monocentric | Prospective, case-control-study | 15 (27%) | 37 (11) | PEM, VGS, MGS, SN, GEN, RBN, | VOG (eyelink II, SR Research |  |
| Huh et al. 2015 [42] | SCA6 | monocentric | Prospective, case-control-study | 11 (45%) | 59 (12) | PEM, rVOR, qHIT, CI | EOG or search coils |  |
| Joiner et al. 2005 [43] | SCA6 | Monocentric | Prospective, case series | 3 (NR) | NR | VGS | VOG (NR) |  |
| Kerber et al. 2005 [44] | SCA1, SCA6, SCA8, LOCA | Monocentric | Prospective, case series | 20 (NR) | NR | PEM, VGS, OKN, rVOR, VORs | EOG |  |
| Kim et al. 2013 [45] | FRDA, SCA1, SCA2, SCA3, SCA6, SCA7, SCA8, other ADCA | monocentric | Prospective, case-control-study | 48 (44%) | 49-57 (5-13) | PEM, VGS, SI, SN, HSN, PN, GEN, | VOR (Micromedical Technologies) |  |
| King et al. 2011 [46] | SCASI | monocentric | Prospective, case-control-study | 2 (NR) | 61 (NR) | PEM, VGS, | Search coils |  |
| Lasker et al. 2005 [47] | SCA6, CA | monocentric | Prospective, case-control-study | 13 (NR | NR | VGS | Search coils |  |
| Lemos et al. 2018 [48] | SCA3 | Monocentric | Prospective, single case report | 1 (0%) | 45 | VGS, SI, GEN, | VOG (VO425, Interacoustics) |  |
| Lewis and Crawford 2002 [49] | AT | Monocentric | Prospective, case series | 3 (NR) | NR | PEM, VGS, | Search coils |  |
| Lewis et al. 1999 [50] | AT | Monocentric | Prospective, case series | 33 (NR) | 10 (NR) | PEM, VGS, SI, OKN, rVOR, | EOG |  |
| Lopez et al. 2019 [51] | FRDA, CA | Monocentric | Prospective, case series | 4 (25%) | 57 (8) | VGS | EOG |  |
| Luis et al. 2016 [52] | FRDA, SCA1, SCA2, SCA3 | Monocentric | Prospective, case-control-study | 30 (50%) | FRDA=36 (12) SCA1=49 (17) SCA2=47 (15) SCA3=50 (12) | qHIT | VOG (eyeseecam, Interacoustics) |  |
| Mariani et al. 2017 [53] | AOA1, AOA2, AT | Monocentric | Prospective, case-control-study | 40 (45%) | AOA1=37 (NR) AOA2=38 (NR) AT=33 (NR) | PEM, VGS, AS, SI, SN, GEN | VOG (SMI) |  |
| Matsuda et al. 2014 [54] | SCA6, SCA31 | monocentric | Prospective, case-control-study | 18 (56%) | 64 (12) | SEM (visual search task) | VOG (Eyelink II, SR research) |  |
| Matsuda et al. 2015 [55] | SCA6, SCA31 | monocentric | Prospective, case-control-study | 19 (53%) | 65 (12) | SEM (visual search task) | VOG (Eyelink II, SR research) | Overlap with Matsuda et al. 2014 [54] |
| Moschner et al. 1994 [56] | FRDA, OPCA, CA | monocentric | Prospective, case-control-study | 71 (45%) | FRDA=21 (10) OPCA=47 (14) CA=43 (15) | PEM, VGS, SI, OKN, rVOR, VORs, SN, GEN | EOG |  |
| Oh et al. 2001 [57] | SCA7 | Monocentric | Prospective, case series | 2 (100%) | 50 (23) | PEM, VGS, rVOR, VORs, GEN | EOG |  |
| Ohyagi et al. 2000 [58] | SCA3 | monocentric | Prospective, case-control-study | 8 (63%) | 36 (14) | NA | NA | Synoptophere used to assess Vergence |
| Pretegiani et al. 2018 [59] | SCA2, LOCA | monocentric | Prospective, case-control-study | 22 (36%) | SCA2=38.7 LOCA=42.4 | AS | VOG (ASL 504 eye tracker) |  |
| Reetz et al. 2018 [60] | SCA2 | monocentric | Prospective, case-control-study | 26 (38%) | 42 (10) | VGS | EOG |  |
| Ribai et al. 2007 [61] | FRDA | monocentric | Prospective, non-randomized treatment study | 37 (NR) | NR | SI | EOG |  |
| Ribeiro et al. 2015 [62] | SCA3 | monocentric | Prospective, case-control-study | 14 (57%) | 42 (8) | oVEMPs, cVEMPs | NA |  |
| Rodríguez-Díaz et al. 2018 [63] | SCA2 | monocentric | Prospective, randomized treatment study | 38 (39%) | 39 (11) | VGS | EOG |  |
| Rodríguez-Labrada et al. 2017 [64] | SCA2 | monocentric | Prospective, case-control-study | 48 (40%) | 41 (10) | VGS | EOG |  |
| Rodríguez-Labrada et al. 2016 [65] | SCA2 | monocentric | Prospective, observational study | 30 (27%) | 40 (11) | VGS | EOG |  |
| Rosini et al. 2013 [66] | Other ADCA | monocentric | Prospective, non-randomized treatment study | 2 (100%) | 59 (1) | VGS, SI | VOG (ASL 504 eye tracker) |  |
| Rufa and Federighi 2011 [67] | SCA2, LOCA | Monocentric | Prospective, case series | 18 (39%) | SCA2=47 LOCA=51 | VGS | VOG (ASL 504 eye tracker) |  |
| Sağlam and Lehnen 2014 [68] | SCA2, LOCA | monocentric | Prospective, case-control-study | 9 (67%) | 57 (13) | qHIT | Search coils |  |
| Seifried et al. 2005 [69] | SCA2 | Monocentric | Prospective, observational study | 82 (37%) | NR | VGS | EOG |  |
| Serrano-Munuera et al. 2013 [70] | SCA37 | monocentric | Prospective, case-control-study | 2 (NR) | 67 (4) | PEM, VGS, OKN | EOG |  |
| Shaikh et al. 2013 [71] | AT | Multicentric | Prospective, case-control-study | 13 (46%) | 26 (NR) | rVOR, rVOR decay Tc, cVEMPs | VOG (SMI) or search coils |  |
| Shaikh et al. 2011 [72] | AT | Multicentric | Prospective, case series | 13 (46%) | 26 (NR) | SI, SN, GEN | VOG (SMI) or search coils |  |
| Shaikh et al. 2009 [73] | AT | Multicentric | Prospective, non-randomized treatment study | 4 (NR) | 35 (14) | rVOR, rVOR decay Tc, SN | Search coils |  |
| Solomon et al. 2005 [74] | NPC | Monocentric | Prospective, single case report | 1 (NR) | NR | VGS | Search coils |  |
| Spieker et al. 1995 [75] | FRDA | Monocentric | Prospective, case-control-study | 13 (38%) | 30 (7) | PEM, VGS, SI, OKN, rVOR, rVOR decay Tc, VORs, SN, GEN, RBN, CI | EOG |  |
| Takegoshi and Murofushi 2000 [76] | SCA3, CA | Monocentric | Prospective, case-control-study | 16 (63%) | 57 (11) | CI, cVEMPs | EOG |  |
| Takeichi et al. 2000 [77] | SCA6 | Monocentric | Prospective, case-control-study | 5 (20%) | 57 (8) | PEM, rVOR, VVOR, VORs, | VOG (unclear) |  |
| Terao et al. 2016 [78] | SCA6, SCA8, SCA31, MSA-C, CA | Monocentric | Prospective, case-control-study | 41 (56%) | SCA=66 (10) MSA-C=63 (8) | VGS, MGS | EOG |  |
| Terao et al. 2017 [79] | SCA6, SCA8, SCA31, MSA-C | Monocentric | Prospective, case-control-study | 44 (NR) | SCD=65 (10) MSA-C=63 (7) | VGS, MGS | EOG |  |
| Velázquez-Pérez et al. 2011 [80] | SCA2 | Monocentric | Prospective, randomized treatment study | 33 (NR) | 42 (6) | VGS | EOG |  |
| Velázquez-Pérez et al. 2012 [81] | SCA2 | Monocentric | Prospective, non-randomized treatment study | 12 (25%) | 38 (9) | VGS | EOG |  |
| Velázquez-Pérez et al. 2014 [82] | SCA2 | Monocentric | Prospective, case-control-study | 37 (65%) | 40 (12) | AS | EOG |  |
| Velázquez-Pérez et al. 2009 [83] | SCA2 | Monocentric | Prospective, case-control-study | 54 (63%) | 36 (NR) | VGS | EOG |  |
| Walterfang et al. 2013 [84] | NPC | Monocentric | Prospective, case-control-study | 10 (40%) | 32 (10) | VGS | VOG (Microguide 1000 infrared limbus) | Same patients as in Walterfang et al. 2013 [84] |
| Walterfang et al. 2012 [85] | NPC | Monocentric | NA, case-control-study | 10 (40%) | 32 (10) | VGS, AS | VOG (Microguide 1000 infrared limbus) |  |
| Wessel et al. 1998 [86] | FRDA, SCA1, SCA3, CA, OPCA | Monocentric | Prospective, case-control-study | 26 (NR) | FRDA=39 (NR) CA=52 (NR) OPCA=43 (NR) | PEM, VGS, SI, OKN, rVOR, rVOR decay Tc, VORs, SN, GEN, RBN, | EOG |  |
| Wiest et al. 2001 [87] | SCA6, EA2 | Monocentric | Prospective, case-control-study | 6 (NR) | 59 (13) | tVOR | Search coils |  |
| Wu et al. 2017 [88] | SCA3 (symptomatic/pre-symptomatic) | Monocentric | Prospective, case-control study | Pre-SCA3=6 (50%)  SCA3=23 (52%) | Pre-SCA3= 29.8±7.4  SCA3= 39.8±10.9 | PEM, VGS, AS, SI, GEN, | VOG (VO425, Interacoustics) |  |
| Yue et al. 1997 [89] | Other ADCA | Monocentric | Prospective, case series | 4 (NR) | NR | PEM, VGS, OKN, rVOR, VORs, | EOG |  |
| Zee et al. 1976 [90] | Other ADCA | Monocentric | Prospective, case series | 12 (63%) | 64 (7) | PEM, VGS, OKN, rVOR, VVOR, VORs, SN, GEN, RBN, | EOG or VOG (custom made) |  |

Abbreviations: ADCA=autosomal-dominant cerebellar ataxia; ARCA=autosomal-recessive cerebellar ataxia; AS=anti-saccades; AT=ataxia telangiectasia; ATLD=ataxia telangiectasia like disease; CA=cerebellar ataxia (not further specified); CI=caloric irrigation; cVEMPs=cervical vestibular-evoked myogenic potentials; EA=episodic ataxia; EOCA=early-onset cerebellar ataxia; EOG=electro-oculography; FRDA=Friedreich Ataxia; GEN=gaze-evoked nystagmus; HN=hyperventilation nystagmus; HSN=head-shaking nystagmus; LOCA=late-onset cerebellar ataxia; MGS=memory-guided saccades; NPC=Niemann Pick disease Type C; OKN=optokinetic nystagmus; OPCA=olivoponto cerebellar ataxia; OVAR=off-vertical axis rotation; oVEMPs=ocular vestibular-evoked myogenic potentials; PEM=pursuit eye movements; PN=positional nystagmus; qHIT=quantitative head-impulse test; RBN=rebound nystagmus; rVOR=rotational vestibulo-ocular reflex; SCA=spinocerebellar ataxia; SEM=saccadic eye movements; SI=saccadic intrusions; SN=spontaneous nystagmus; SVV=subjective visual vertical; tVOR=translational vestibulo-ocular reflex; VOG=video-oculography; VORs=vestibulo-ocular reflex suppression; VGS=visually-guided saccades; VVOR=visually-enhanced vestibulo-ocular reflex;

| **Table S3: Diagnostic effects observed across studies for other oculomotor parameters.** | | | | | | | | | | | | |
| --- | --- | --- | --- | --- | --- | --- | --- | --- | --- | --- | --- | --- |
|  | **OKN** | | **Vestibulo-ocular reflex (VOR)** | | | | **RBN** | **Positional nystagmus** | | **Vestibular responses** | | |
|  | Micro-opsoclonus (frequency) | gain | VOR gain | VVOR gain | VORS preserved | VOR decay Tc | RBN (presence) | pDBN (presence) | HSN (presence) | Caloric irrigation (SPV) | oVEMPs | cVEMPs |
| **FRDA [2, 6, 19, 26, 28, 29, 32, 33, 52, 56, 61, 75, 86]** |  | → / ↓ / ↓↓ | ↓↓ | ↓↓ | → / ↓ | ↓↓ | 13-15% |  |  | ↓↓ / ↓↓↓ |  |  |
| **SCA1 [2, 12, 14, 44, 45, 52]** |  | ↓↓ | →/↓ |  |  |  | 33% |  | + (hor) |  |  |  |
| **SCA2 [14, 30, 45, 52, 59, 60, 64, 65, 67, 69, 82, 83]** |  | ↓ | → |  |  |  |  | (+) | ∅ |  |  |  |
| **SCA3 [2, 14, 15, 27, 34, 35, 37, 45, 48, 52, 62, 76, 88]** | + | ↓↓ | ↓↓ |  |  |  | 100% |  | + (hor) |  | ∅ (61%) | ∅ (10-67%) |
| **SCA6 [9, 14, 18, 36, 42, 44, 45, 54, 77]** |  | ↓↓↓ | → / ↑ |  | ↓↓ |  | 40% | 57% | 83% (vert) |  |  |  |
| **SCA17 [41]** |  |  |  |  |  |  |  |  |  |  |  |  |
| **A-T [7, 49, 50, 53, 72, 73]** |  | ↓↓ | → / ↑ |  | ↓↓ | → |  |  |  |  |  | ↑ (amp) |
| **NPC [10, 11, 38, 74]** |  |  |  |  |  |  |  |  |  | → | → | → |
| **CANVAS / RFC-1 [22, 91-94]** |  |  | ↓↓ |  | ↓↓ | → | 9% |  |  | ↓↓ | ↓ (amp; 25%) | ∅ (100%) |

Symbol legend: ∅=finding absent; (+)/+/++/+++=finding present rarely ((+)) / sometimes (+) / in a significant fraction (++) / frequently (+++); ↑/↑↑/↑↑↑ = value increased mildly (↑) / moderately (↑↑) / strongly (↑↑↑); → = value unchanged, ↓/↓↓/↓↓↓ = value decreased mildly (↓) / moderately (↓↓) / strongly (↓↓↓) compared to healthy controls;

Abbreviations: amp=amplitude; A-T=ataxia telangiectasia; CANVAS=cerebellar ataxia-nystagmus-vestibular areflexia syndrome; FRDA=Friedreich ataxia; HSN=head shaking nystagmus; NPC=Niemann-Pick disease type C; OKN=optokinetic nystagmus; pDBN=positional downbeat nystagmus; RBN=rebound nystagmus; RFC-1=replication factor complex subunit 1; SCA=spinocerebellar ataxia; vert=vertical; Tc=time constant; VOR=vestibulo-ocular reflex; VVOR=visually-enhanced VOR; VORS=vestibulo-ocular reflex suppression.

Table S4: characteristics of eye movement recordings

| **Table S4: characteristics of eye movement recordings** | | |
| --- | --- | --- |
|  |  |  |
| **Parameter** | **Studies (n)** | **Subjects (n)** |
| *Pursuit eye movements* | 33 | 637 |
| *Saccadic eye movements* |  |  |
| visually-guided SEM | 61 | 1172 |
| memory-guided SEM | 4 | 72 |
| antisaccades | 8 | 194 |
| other | 2 | 19 |
| *Saccadic intrusions* | 25 | 502 |
| *OKN* | 15 | 270 |
| *gaze holding* |  |  |
| spontaneous nystagmus | 18 | 411 |
| gaze-evoked nystagmus | 23 | 494 |
| rebound nystagmus | 9 | 129 |
| *Triggered nystagmus* |  |  |
| head-shaking nystagmus | 1 | 48 |
| hyperventilation-induced nystagmus | 1 | 11 |
| positional nystagmus | 2 | 63 |
| *VOR* |  |  |
| VOR rotational | 20 | 301 |
| VOR decay Tc | 6 | 55 |
| VVOR rotational | 4 | 61 |
| VOR translational | 2 | 17 |
| OVAR | 1 | 10 |
| VOR suppression | 13 | 226 |
| qHIT | 8 | 169 |
| Caloric irrigation | 7 | 74 |
| *VEMPs* |  |  |
| oVEMPs | 2 | 17 |
| cVEMPs | 5 | 54 |
| *behavioral tasks* |  |  |
| subjective visual vertical (SVV) | 2 | 18 |

Abbreviations: cVEMPS=cervical vestibular-evoked myogenic potentials; OKN=optokinetic nystagmus; OVAR=off-vertical axis rotation; oVEMPS=ocular vestibular-evoked myogenic potentials; qHIT=quantitative head-impulse test; SEM=saccadic eye movements; Tc=time constant; VOR=vestibulo-ocular reflex; VVOR=visually-enhanced VOR; VORS=vestibulo-ocular reflex suppression.

Supplementary Table 5 – significant correlation analyses in distinct hereditary ataxias

| **Table S5: significant correlations in distinct hereditary ataxias** | | | | | | |
| --- | --- | --- | --- | --- | --- | --- |
|  |  |  |  |  |  |  |
| **Parameter used for correlation analysis at study-level** | **Studies (n, [fraction of studies reporting significant correlations])** | **Subjects (n)** |  |  |  |  |
| clinical scores | 18 (78%) | 417 |  |  |  |  |
| other clinical parameters | 17 (59%) | 431 |  |  |  |  |
| Imaging parameters | 6 (83%) | 115 |  |  |  |  |
| Genetic testing | 9 (67%) | 283 |  |  |  |  |
| Patient-reported outcomes | 1 (0%) | 0 |  |  |  |  |
| Any parameter | 29 (76%) | 653 |  |  |  |  |
|  |  |  |  |  |  |  |
| **Number of significant correlations reported** |  |  |  |  |  |  |
|  | **PEM** | **SEM** | **SI** | **SN** | **GEN** | **qHIT** |
| **FRDA [29, 32, 39, 40]** |  | 17 | 2 |  |  |  |
| **SCA2 [30, 60, 64, 65, 69, 82, 83]** |  | 17 |  |  |  |  |
| **SCA3 [27, 88]** | 6 | 19 | 2 | 4 | 11 | 7 |
| **SCA6 [42]** |  |  |  |  |  | 1 |
| **SCA17 [41]** | 3 | 1 |  |  |  |  |
| **A-T [50]** | 2 |  |  |  |  |  |
| **NPC [38, 84, 85]** |  | 12 |  |  |  |  |
| **ADCA [13]** |  | 1 |  |  |  |  |
| ***all hereditary ataxia syndromes*** | 11 | 67 | 4 | 4 | 11 | 8 |

§ This included FRDA, SCA1 and SCA3 patients [2]

° This included FRDA, SCA1, SCA2 and SCA3 patients [52]

Abbreviations: A-T=ataxia telangiectasia; ADCA=autosomal-dominant cerebellar ataxia; FRDA=Friedreich ataxia; GEN=gaze-evoked nystagmus; NPC=Niemann-Pick disease type C; PEM=pursuit eye movements; qHIT=quantitative head-impulse test; SCA=spinocerebellar ataxia; SEM=saccadic eye movements; SI=saccadic intrusions; SN=spontaneous nystagmus;

Supplementary Table 6: Detailed correlation results between oculomotor testing and other clinical information. For each diagnosis and eye movement paradigm, the two variables included in the correlation are indicated along with the level of the correlation strength.

| **Table S6: detailed correlation analyses – oculomotor testing and other parameters in hereditary ataxias (significant correlations only)** | | | | | | | | | | | |
| --- | --- | --- | --- | --- | --- | --- | --- | --- | --- | --- | --- |
|  | **SEM** | | | | | | | | **Saccadic intrusions** | | |
| **FRDA** | ***FARS and SLCLC:***   - VGS and AS latency*** [32], VGS latency**-*** [39]   ***FARS***:   - horizontal latency** [29], AS and MGS error latency** [32], VG latency**-*** [40] | | | | ***SLCLC:***   - horizontal latency*** [29], AS error latency** [32], MGS latency*** [32], VGS latency*** [40]   ***disease duration:***   - AS latency** [32], MGS error (proportion)*** [32]   ***GAA repeat length:***   - MGS errors (proportion) ** [32] | | | | ***SLCLC:***   - Macro-SWJ frequency *** [29]   ***age at sx onset***   - Macro-SWJ frequency ** [29] | | |
|  | ***SEM*** | | | | | | | | | | |
| **SCA2** | ***ICARS:***   - mean velocity and duration (strength NR) [30]   ***SARA:***   - Horizontal PV* [64]   ***disease duration:***   - PV (strength NR) [69]   ***Age:***   - Horizontal PV** [64]   ***age at sx onset:***   - Horizontal PV** [64], PV (strength NR) [69] | | | | | ***Pontine volumes:***   - Horizontal PV (preclinical SCA2)** [60] - Horizontal PV (manifest SCA2)* [60]   ***Anterior–posterior diameter of pontine brainstem:***   - Horizontal PV (preclinical SCA2)** [60] - Horizontal PV (manifest SCA2)* [60]   ***CAG repeat length:***   - Horizontal PV** [64] - Progression rate of horizontal PV*** [65] - Horizontal PV (strength NR) [69] | | | *For pre-ataxic carriers*:  ***time to sx onset:***   - Antisaccade latencies** [82] - Horizontal PV* [83]   ***CAG repeat length:***   - Horizontal PV* [83] | | |
|  | **PEM** | **SEM** | | | | | | **SN / GEN / Saccadic intrusions** | | **qHIT** | |
| **SCA3** | *Pre-ataxic carriers & ataxic patients*:  ***ICARS / SCAFI / CCFS:***   - vertical gain* [27]   ***SARA***:   - Horizontal** and vertical* gain [88]   ***disease duration:***   - Horizontal gain** [88] | *For pre-ataxic carriers*:  ***SARA***:   - reflexive vertical saccades slope*** [27]   ***time to sx onset:***   - vertical saccades slope** [27]   *Pre-ataxic carriers & ataxic patients*:  ***ICARS / NESSCA:***   - vertical saccades slope** [27]   ***INAScount / SCAFI / CCFS:***   - vertical saccades slope* [27]   ***SARA***:   - Upward saccade latency* [88] - Horizontal* and upward* PV [88] - Horizontal** and upward** saccadic accuracy [88] - Antisaccade error rate** [88]   ***disease duration:***   - Upward saccade latency* [88] - Upward* PV [88] - Horizontal* and upward** saccadic accuracy [88] - Antisaccade error rate* [88]   ***GAC repeat length***:   - Antisaccade error rate* [88] | | | | | | *For pre-ataxic carriers*:  ***SARA:***   - SPV-GE*** [27]   ***time to sx onset***   - SPV-GE** [27]   *Pre-ataxic carriers & ataxic patients*:  ***ICARS / NESSCA / SCAFI / CCFS***   - Horizontal SPV central nystagmus* [27]   ***ICARS / NESSCA / INAScount / SCAFI / CCFS***   - SPV-GE*** [27]   ***SARA***:   - Frequency** and amplitude*** of horizontal GEN [88] - Frequency* and amplitude* of SWJ [88]   ***disease duration:***   - Frequency** and amplitude** of horizontal GEN [88] | | *For pre-ataxic carriers*:  ***SARA:***   - Horizontal VORr gain*** [27]   ***time to sx onset***   - Horizontal VORr gain** [27]   *Pre-ataxic carriers & ataxic patients*:  ***ICARS / NESSCA***   - Horizontal VORr gain*** [27]   ***INAScount / SCAFI / CCFS***   - Horizontal VORr gain** [27] | |
|  | **qHIT** | | | | | | | | | | |
| **SCA6** | ***ICARS***   - HC qHIT gain*** [42] | | | | | | | | | | |
|  | **PEM** | | | | | | | | | **SEM** | |
| **SCA17** | ***ICARS***:   - Horizontal gain*** [41] - latency of initial horizontal response (step ramp)** [41] | | | | | | ***Disease duration:***   - Horizontal gain*** [41] | | | ***Disease duration:***   - Horizontal gain*** [41] | |
|  | ***PEM*** | | | | | | | | | | |
| **AT** | ***A-T index:***   - Horizontal gain** [50] | | | ***Age***:   - Horizontal gain** [50] | | | | | | | |
|  | ***SEM*** | | | | | | | | | | |
| **NPC** | ***Total cerebellar volume***   - Horizontal self-paced SEM (gain)*** [84]   ***Total cerebellar grey matter***   - Horizontal self-paced SEM (gain)*** [84] | | ***Midbrain midsagittal area***   - Horizontal SEM (PV, gain)** [85] - Horizontal self-paced SEM (n) ** [85]   ***Pontine-midbrain-ratio:***   - Horizontal SEM (PV, gain)*** [85] - Horizontal self-paced SEM (PV, gain)** [85] | | | | | ***averaged pRNFL***   - upward vertical saccade amp*** [38] - Horizontal PV *** [38] - Mean duration of horizontal saccades*** [38] | | | ***averaged GCIP***   - upward vertical saccade amp*** [38] - PV horizontal saccades*** [38] - vertical gain*** [38] |

Strength of correlation (Pearson or Spearman): strong (***), moderate (**), weak (*). SEM refer to visually-guided saccade paradigm unless otherwise stated.

Abbrevations: AS=antisaccades; CCFS= Composite Cerebellar Functional Score; FARS= Friedreich Ataxia Rating Scale; GCIP= combined ganglion cell and inner plexiform layer; GEN=gaze-evoked nystagmus; HC=horizontal canal; ICARS= International Cooperative Ataxia Rating Scale; MGS=memory-guided saccades; NESSCA= Neurological Examination Score for Spinocerebellar Ataxia; NR=not reported; PEM=pursuit eye movements; pRNFL= peripapillary retinal nerve fiber layer thickness; PV=peak velocity; qHIT=quantitative head-impulse test; SARA= Scale for the Rating and Assessment of Ataxia; SCAFI= Spinocerebellar Ataxia Functional Index; INAScount= Inventory of Non-Ataxia Signs; SEM=saccadic eye movements; SLCLC=Sloan Low Contrast Letter Chart; SN=spontaneous nystagmus; SPV= slow phase velocity; sx=symptom; SWJ=square-wave jerks; VGS=visually-guided saccades.

## References

[1] Cohen J. A coefficient for agreement for nominal scales. Educ Psychol Meas 1960: 20:37-46.

[2] Alexandre MF, Rivaud-Péchoux S, Challe G, Durr A and Gaymard B. Functional consequences of oculomotor disorders in hereditary cerebellar ataxias. Cerebellum (London, England) 2013: 12:396-405. doi 10.1007/s12311-012-0433-z

[3] Anastasopoulos D, Haslwanter T, Fetter M and Dichgans J. Smooth pursuit eye movements and otolith-ocular responses are differently impaired in cerebellar ataxia. Brain : a journal of neurology 1998: 121 ( Pt 8):1497-505. doi 10.1093/brain/121.8.1497

[4] Anderson JH, Christova PS, Xie TD, Schott KS, Ward K and Gomez CM. Spinocerebellar ataxia in monozygotic twins. Archives of neurology 2002: 59:1945-51. doi 10.1001/archneur.59.12.1945

[5] Anderson JH, Yavuz MC, Kazar BM, Christova P and Gomez CM. The vestibulo-ocular reflex and velocity storage in spinocerebellar ataxia 8. Archives italiennes de biologie 2002: 140:323-9.

[6] Baloh RW, Konrad HR and Honrubia V. Vestibulo-ocular function in patients with cerebellar atrophy. Neurology 1975: 25:160-8. doi 10.1212/wnl.25.2.160

[7] Baloh RW, Yee RD and Boder E. Eye movements in ataxia-telangiectasia. Neurology 1978: 28:1099-104. doi 10.1212/wnl.28.11.1099

[8] Bargagli A, Rosini F, Zanca D, Serchi V and Rufa A. Ataxia with oculomotor apraxia type 2 (AOA2): an eye movement study of two siblings. Neurological sciences : official journal of the Italian Neurological Society and of the Italian Society of Clinical Neurophysiology 2021: 42:3039-42. doi 10.1007/s10072-021-05206-1

[9] Bour LJ, van Rootselaar AF, Koelman JH and Tijssen MA. Oculomotor abnormalities in myoclonic tremor: a comparison with spinocerebellar ataxia type 6. Brain : a journal of neurology 2008: 131:2295-303. doi 10.1093/brain/awn177

[10] Bremova T, Krafczyk S, Bardins S, Reinke J and Strupp M. Vestibular function in patients with Niemann-Pick type C disease. Journal of neurology 2016: 263:2260-70. doi 10.1007/s00415-016-8247-4

[11] Bremova T, Malinova V, Amraoui Y, Mengel E, Reinke J, Kolnikova M and Strupp M. Acetyl-dl-leucine in Niemann-Pick type C: A case series. Neurology 2015: 85:1368-75. doi 10.1212/WNL.0000000000002041

[12] Bürk K, Abele M, Fetter M, Dichgans J, Skalej M, Laccone F, Didierjean O, Brice A and Klockgether T. Autosomal dominant cerebellar ataxia type I clinical features and MRI in families with SCA1, SCA2 and SCA3. Brain : a journal of neurology 1996: 119 ( Pt 5):1497-505. doi 10.1093/brain/119.5.1497

[13] Bürk K, Fetter M, Skalej M, Laccone F, Stevanin G, Dichgans J and Klockgether T. Saccade velocity in idiopathic and autosomal dominant cerebellar ataxia. Journal of neurology, neurosurgery, and psychiatry 1997: 62:662-4. doi 10.1136/jnnp.62.6.662

[14] Buttner N, Geschwind D, Jen JC, Perlman S, Pulst SM and Baloh RW. Oculomotor phenotypes in autosomal dominant ataxias. Archives of neurology 1998: 55:1353-7. doi 10.1001/archneur.55.10.1353

[15] Caspi A, Zivotofsky AZ and Gordon CR. Multiple saccadic abnormalities in spinocerebellar ataxia type 3 can be linked to a single deficiency in velocity feedback. Investigative ophthalmology & visual science 2013: 54:731-8. doi 10.1167/iovs.12-10689

[16] Chang Z, Chen Z, Stephen CD, Schmahmann JD, Wu HT, Sapiro G and Gupta AS. Accurate detection of cerebellar smooth pursuit eye movement abnormalities via mobile phone video and machine learning. Scientific reports 2020: 10:18641. doi 10.1038/s41598-020-75661-x

[17] Choi JH, Seo JD, Choi YR, Kim MJ, Shin JH, Kim JS and Choi KD. Exercise-induced downbeat nystagmus in a Korean family with a nonsense mutation in CACNA1A. Neurological sciences : official journal of the Italian Neurological Society and of the Italian Society of Clinical Neurophysiology 2015: 36:1393-6. doi 10.1007/s10072-015-2157-6

[18] Christova P, Anderson JH and Gomez CM. Impaired eye movements in presymptomatic spinocerebellar ataxia type 6. Archives of neurology 2008: 65:530-6. doi 10.1001/archneur.65.4.530

[19] Ciuffreda KJ, Kenyon RV and Stark L. Eye movements during reading: further case reports. American journal of optometry and physiological optics 1985: 62:844-52. doi 10.1097/00006324-198512000-00005

[20] Clausi S, De Luca M, Chiricozzi FR, Tedesco AM, Casali C, Molinari M and Leggio MG. Oculomotor deficits affect neuropsychological performance in oculomotor apraxia type 2. Cortex; a journal devoted to the study of the nervous system and behavior 2013: 49:691-701. doi 10.1016/j.cortex.2012.02.007

[21] Coin JT and Vance JM. Gabapentin Relieves Vertigo of Periodic Vestibulocerebellar Ataxia: 3 Cases and Possible Mechanism. Movement disorders : official journal of the Movement Disorder Society 2021. doi 10.1002/mds.28491

[22] Costales M, Casanueva R, Suárez V, Asensi JM, Cifuentes GA, Diñeiro M, Cadiñanos J, López F, Álvarez-Marcos C, Otero A, Gómez J, Llorente JL and Cabanillas R. CANVAS: A New Genetic Entity in the Otorhinolaryngologist's Differential Diagnosis. Otolaryngology--head and neck surgery : official journal of American Academy of Otolaryngology-Head and Neck Surgery 2021:1945998211008398. doi 10.1177/01945998211008398

[23] Crane BT, Tian JR and Demer JL. Initial vestibulo-ocular reflex during transient angular and linear acceleration in human cerebellar dysfunction. Experimental brain research 2000: 130:486-96. doi 10.1007/s002219900266

[24] Crowdy KA, Hollands MA, Ferguson IT and Marple-Horvat DE. Evidence for interactive locomotor and oculomotor deficits in cerebellar patients during visually guided stepping. Experimental brain research 2000: 135:437-54. doi 10.1007/s002210000539

[25] Dakin CJ, Peters A, Giunti P and Day BL. Cerebellar Degeneration Increases Visual Influence on Dynamic Estimates of Verticality. Current biology : CB 2018: 28:3589-98.e3. doi 10.1016/j.cub.2018.09.049

[26] Dale RT, Kirby AW and Jampel RS. Square wave jerks in Friedreich's ataxia. American journal of ophthalmology 1978: 85:400-6. doi 10.1016/s0002-9394(14)77738-4

[27] de Oliveira CM, Leotti VB, Bolzan G, Cappelli AH, Rocha AG, Ecco G, Kersting N, Rieck M, Martins AC, Sena LS, Saraiva-Pereira ML and Jardim LB. Pre-ataxic Changes of Clinical Scales and Eye Movement in Machado-Joseph Disease: BIGPRO Study. Movement disorders : official journal of the Movement Disorder Society 2021. doi 10.1002/mds.28466

[28] Ell J, Prasher D and Rudge P. Neuro-otological abnormalities in Friedreich's ataxia. Journal of neurology, neurosurgery, and psychiatry 1984: 47:26-32. doi 10.1136/jnnp.47.1.26

[29] Fahey MC, Cremer PD, Aw ST, Millist L, Todd MJ, White OB, Halmagyi M, Corben LA, Collins V, Churchyard AJ, Tan K, Kowal L and Delatycki MB. Vestibular, saccadic and fixation abnormalities in genetically confirmed Friedreich ataxia. Brain : a journal of neurology 2008: 131:1035-45. doi 10.1093/brain/awm323

[30] Federighi P, Cevenini G, Dotti MT, Rosini F, Pretegiani E, Federico A and Rufa A. Differences in saccade dynamics between spinocerebellar ataxia 2 and late-onset cerebellar ataxias. Brain : a journal of neurology 2011: 134:879-91. doi 10.1093/brain/awr009

[31] Federighi P, Ramat S, Rosini F, Pretegiani E, Federico A and Rufa A. Characteristic Eye Movements in Ataxia-Telangiectasia-Like Disorder: An Explanatory Hypothesis. Frontiers in neurology 2017: 8:596. doi 10.3389/fneur.2017.00596

[32] Fielding J, Corben L, Cremer P, Millist L, White O and Delatycki M. Disruption to higher order processes in Friedreich ataxia. Neuropsychologia 2010: 48:235-42. doi 10.1016/j.neuropsychologia.2009.09.009

[33] Furman JM, Perlman S and Baloh RW. Eye movements in Friedreich's ataxia. Archives of neurology 1983: 40:343-6. doi 10.1001/archneur.1983.04050060043006

[34] Geisinger D, Elyoseph Z, Zaltzman R, Mintz M and Gordon CR. Angular vestibulo ocular reflex loss with preserved saccular function in Machado-Joseph disease. Journal of the neurological sciences 2021: 424:117393. doi 10.1016/j.jns.2021.117393

[35] Ghasia FF, Wilmot G, Ahmed A and Shaikh AG. Strabismus and Micro-Opsoclonus in Machado-Joseph Disease. Cerebellum (London, England) 2016: 15:491-7. doi 10.1007/s12311-015-0718-0

[36] Gomez CM, Thompson RM, Gammack JT, Perlman SL, Dobyns WB, Truwit CL, Zee DS, Clark HB and Anderson JH. Spinocerebellar ataxia type 6: gaze-evoked and vertical nystagmus, Purkinje cell degeneration, and variable age of onset. Annals of neurology 1997: 42:933-50. doi 10.1002/ana.410420616

[37] Gordon CR, Zivotofsky AZ and Caspi A. Impaired vestibulo-ocular reflex (VOR) in spinocerebellar ataxia type 3 (SCA3): bedside and search coil evaluation. Journal of vestibular research : equilibrium & orientation 2014: 24:351-5. doi 10.3233/ves-140527

[38] Havla J, Moser M, Sztatecsny C, Lotz-Havla AS, Maier EM, Hizli B, Schinner R, Kümpfel T, Strupp M, Bremova-Ertl T and Schneider SA. Retinal axonal degeneration in Niemann-Pick type C disease. Journal of neurology 2020: 267:2070-82. doi 10.1007/s00415-020-09796-2

[39] Hocking DR, Corben LA, Fielding J, Cremer PD, Millist L, White OB and Delatycki MB. Saccade reprogramming in Friedreich ataxia reveals impairments in the cognitive control of saccadic eye movement. Brain and cognition 2014: 87:161-7. doi 10.1016/j.bandc.2014.03.018

[40] Hocking DR, Fielding J, Corben LA, Cremer PD, Millist L, White OB and Delatycki MB. Ocular motor fixation deficits in Friedreich ataxia. Cerebellum (London, England) 2010: 9:411-8. doi 10.1007/s12311-010-0178-5

[41] Hübner J, Sprenger A, Klein C, Hagenah J, Rambold H, Zühlke C, Kömpf D, Rolfs A, Kimmig H and Helmchen C. Eye movement abnormalities in spinocerebellar ataxia type 17 (SCA17). Neurology 2007: 69:1160-8. doi 10.1212/01.wnl.0000276958.91986.89

[42] Huh YE, Kim JS, Kim HJ, Park SH, Jeon BS, Kim JM, Cho JW and Zee DS. Vestibular Performance During High-Acceleration Stimuli Correlates with Clinical Decline in SCA6. Cerebellum (London, England) 2015: 14:284-91. doi 10.1007/s12311-015-0650-3

[43] Joiner WM, Shelhamer M and Ying SH. Cerebellar influence in oculomotor phase-transition behavior. Annals of the New York Academy of Sciences 2005: 1039:536-9. doi 10.1196/annals.1325.062

[44] Kerber KA, Jen JC, Perlman S and Baloh RW. Late-onset pure cerebellar ataxia: differentiating those with and without identifiable mutations. Journal of the neurological sciences 2005: 238:41-5. doi 10.1016/j.jns.2005.06.006

[45] Kim JS, Kim JS, Youn J, Seo DW, Jeong Y, Kang JH, Park JH and Cho JW. Ocular motor characteristics of different subtypes of spinocerebellar ataxia: distinguishing features. Movement disorders : official journal of the Movement Disorder Society 2013: 28:1271-7. doi 10.1002/mds.25464

[46] King SA, Schneider RM, Serra A and Leigh RJ. Critical role of cerebellar fastigial nucleus in programming sequences of saccades. Annals of the New York Academy of Sciences 2011: 1233:155-61. doi 10.1111/j.1749-6632.2011.06119.x

[47] Lasker AG, Isotalo EH and Zee DS. Predictive saccades to a regularly alternating target in cerebellar patients. Annals of the New York Academy of Sciences 2005: 1039:544-7. doi 10.1196/annals.1325.064

[48] Lemos J, Novo A, Duque C, Castelhano J, Eggenberger E and Januário C. "Pinball" intrusions in spinocerebellar ataxia type 3. Neurology 2018: 90:36-7. doi 10.1212/wnl.0000000000004772

[49] Lewis RF and Crawford TO. Slow target-directed eye movements in ataxia-telangiectasia. Investigative ophthalmology & visual science 2002: 43:686-91.

[50] Lewis RF, Lederman HM and Crawford TO. Ocular motor abnormalities in ataxia telangiectasia. Annals of neurology 1999: 46:287-95. doi 10.1002/1531-8249(199909)46:3<287::aid-ana3>3.0.co;2-0

[51] López A, Ferrero F and Postolache O. An Affordable Method for Evaluation of Ataxic Disorders Based on Electrooculography. Sensors (Basel, Switzerland) 2019: 19. doi 10.3390/s19173756

[52] Luis L, Costa J, Munoz E, de Carvalho M, Carmona S, Schneider E, Gordon CR and Valls-Sole J. Vestibulo-ocular reflex dynamics with head-impulses discriminates spinocerebellar ataxias types 1, 2 and 3 and Friedreich ataxia. Journal of vestibular research : equilibrium & orientation 2016: 26:327-34. doi 10.3233/VES-160579

[53] Mariani LL, Rivaud-Pechoux S, Charles P, Ewenczyk C, Meneret A, Monga BB, Fleury MC, Hainque E, Maisonobe T, Degos B, Echaniz-Laguna A, Renaud M, Wirth T, Grabli D, Brice A, Vidailhet M, Stoppa-Lyonnet D, Dubois-d'Enghien C, Le Ber I, Koenig M, Roze E, Tranchant C, Durr A, Gaymard B and Anheim M. Comparing ataxias with oculomotor apraxia: a multimodal study of AOA1, AOA2 and AT focusing on video-oculography and alpha-fetoprotein. Scientific reports 2017: 7:15284. doi 10.1038/s41598-017-15127-9

[54] Matsuda S, Matsumoto H, Furubayashi T, Fukuda H, Emoto M, Hanajima R, Tsuji S, Ugawa Y and Terao Y. Top-down but not bottom-up visual scanning is affected in hereditary pure cerebellar ataxia. PloS one 2014: 9:e116181. doi 10.1371/journal.pone.0116181

[55] Matsuda S, Matsumoto H, Furubayashi T, Fukuda H, Hanajima R, Tsuji S, Ugawa Y and Terao Y. Visual scanning area is abnormally enlarged in hereditary pure cerebellar ataxia. Cerebellum (London, England) 2015: 14:63-71. doi 10.1007/s12311-014-0600-5

[56] Moschner C, Perlman S and Baloh RW. Comparison of oculomotor findings in the progressive ataxia syndromes. Brain : a journal of neurology 1994: 117 ( Pt 1):15-25. doi 10.1093/brain/117.1.15

[57] Oh AK, Jacobson KM, Jen JC and Baloh RW. Slowing of voluntary and involuntary saccades: an early sign in spinocerebellar ataxia type 7. Annals of neurology 2001: 49:801-4. doi 10.1002/ana.1059

[58] Ohyagi Y, Yamada T, Okayama A, Sakae N, Yamasaki T, Ohshima T, Sakamoto T, Fujii N and Kira J. Vergence disorders in patients with spinocerebellar ataxia 3/Machado-Joseph disease: a synoptophore study. Journal of the neurological sciences 2000: 173:120-3. doi 10.1016/s0022-510x(99)00309-3

[59] Pretegiani E, Piu P, Rosini F, Federighi P, Serchi V, Tumminelli G, Dotti MT, Federico A and Rufa A. Anti-Saccades in Cerebellar Ataxias Reveal a Contribution of the Cerebellum in Executive Functions. Frontiers in neurology 2018: 9:274. doi 10.3389/fneur.2018.00274

[60] Reetz K, Rodríguez-Labrada R, Dogan I, Mirzazade S, Romanzetti S, Schulz JB, Cruz-Rivas EM, Alvarez-Cuesta JA, Aguilera Rodríguez R, Gonzalez Zaldivar Y, Auburger G and Velázquez-Pérez L. Brain atrophy measures in preclinical and manifest spinocerebellar ataxia type 2. Annals of clinical and translational neurology 2018: 5:128-37. doi 10.1002/acn3.504

[61] Ribaï P, Pousset F, Tanguy ML, Rivaud-Pechoux S, Le Ber I, Gasparini F, Charles P, Béraud AS, Schmitt M, Koenig M, Mallet A, Brice A and Dürr A. Neurological, cardiological, and oculomotor progression in 104 patients with Friedreich ataxia during long-term follow-up. Archives of neurology 2007: 64:558-64. doi 10.1001/archneur.64.4.558

[62] Ribeiro RS, Pereira MM, Pedroso JL, Braga-Neto P, Barsottini OG and Manzano GM. Cervical and ocular vestibular evoked potentials in Machado-Joseph disease: Functional involvement of otolith pathways. Journal of the neurological sciences 2015: 358:294-8. doi 10.1016/j.jns.2015.09.013

[63] Rodríguez-Díaz JC, Velázquez-Pérez L, Rodríguez Labrada R, Aguilera Rodríguez R, Laffita Pérez D, Canales Ochoa N, Medrano Montero J, Estupiñán Rodríguez A, Osorio Borjas M, Góngora Marrero M, Reynaldo Cejas L, González Zaldivar Y and Almaguer Gotay D. Neurorehabilitation therapy in spinocerebellar ataxia type 2: A 24-week, rater-blinded, randomized, controlled trial. Movement disorders : official journal of the Movement Disorder Society 2018: 33:1481-7. doi 10.1002/mds.27437

[64] Rodríguez-Labrada R, Vázquez-Mojena Y, Canales-Ochoa N, Medrano-Montero J and Velázquez-Pérez L. Heritability of saccadic eye movements in spinocerebellar ataxia type 2: insights into an endophenotype marker. Cerebellum & ataxias 2017: 4:19. doi 10.1186/s40673-017-0078-2

[65] Rodríguez-Labrada R, Velázquez-Pérez L, Auburger G, Ziemann U, Canales-Ochoa N, Medrano-Montero J, Vázquez-Mojena Y and González-Zaldivar Y. Spinocerebellar ataxia type 2: Measures of saccade changes improve power for clinical trials. Movement disorders : official journal of the Movement Disorder Society 2016: 31:570-8. doi 10.1002/mds.26532

[66] Rosini F, Federighi P, Pretegiani E, Piu P, Leigh RJ, Serra A, Federico A and Rufa A. Ocular-motor profile and effects of memantine in a familial form of adult cerebellar ataxia with slow saccades and square wave saccadic intrusions. PloS one 2013: 8:e69522. doi 10.1371/journal.pone.0069522

[67] Rufa A and Federighi P. Fast versus slow: different saccadic behavior in cerebellar ataxias. Annals of the New York Academy of Sciences 2011: 1233:148-54. doi 10.1111/j.1749-6632.2011.06126.x

[68] Saglam M and Lehnen N. Gaze stabilization in chronic vestibular-loss and in cerebellar ataxia: interactions of feedforward and sensory feedback mechanisms. Journal of vestibular research : equilibrium & orientation 2014: 24:425-31. doi 10.3233/VES-140538

[69] Seifried C, Velázquez-Pérez L, Santos-Falcón N, Abele M, Ziemann U, Almaguer LE, Martínez-Góngora E, Sánchez-Cruz G, Canales N, Pérez-González R, Velázquez-Manresa M, Viebahn B, Stuckrad-Barre S, Klockgether T, Fetter M and Auburger G. Saccade velocity as a surrogate disease marker in spinocerebellar ataxia type 2. Annals of the New York Academy of Sciences 2005: 1039:524-7. doi 10.1196/annals.1325.059

[70] Serrano-Munuera C, Corral-Juan M, Stevanin G, San Nicolás H, Roig C, Corral J, Campos B, de Jorge L, Morcillo-Suárez C, Navarro A, Forlani S, Durr A, Kulisevsky J, Brice A, Sánchez I, Volpini V and Matilla-Dueñas A. New subtype of spinocerebellar ataxia with altered vertical eye movements mapping to chromosome 1p32. JAMA neurology 2013: 70:764-71. doi 10.1001/jamaneurol.2013.2311

[71] Shaikh AG, Marti S, Tarnutzer AA, Palla A, Crawford TO, Zee DS and Straumann D. Effects of 4-aminopyridine on nystagmus and vestibulo-ocular reflex in ataxia-telangiectasia. Journal of neurology 2013: 260:2728-35. doi 10.1007/s00415-013-7046-4

[72] Shaikh AG, Marti S, Tarnutzer AA, Palla A, Crawford TO, Straumann D, Carey JP, Nguyen KD and Zee DS. Ataxia telangiectasia: a "disease model" to understand the cerebellar control of vestibular reflexes. Journal of neurophysiology 2011: 105:3034-41. doi 10.1152/jn.00721.2010

[73] Shaikh AG, Marti S, Tarnutzer AA, Palla A, Crawford TO, Straumann D, Taylor AM and Zee DS. Gaze fixation deficits and their implication in ataxia-telangiectasia. Journal of neurology, neurosurgery, and psychiatry 2009: 80:858-64. doi 10.1136/jnnp.2008.170522

[74] Solomon D, Winkelman AC, Zee DS, Gray L and Büttner-Ennever J. Niemann-Pick type C disease in two affected sisters: ocular motor recordings and brain-stem neuropathology. Annals of the New York Academy of Sciences 2005: 1039:436-45. doi 10.1196/annals.1325.041

[75] Spieker S, Schulz JB, Petersen D, Fetter M, Klockgether T and Dichgans J. Fixation instability and oculomotor abnormalities in Friedreich's ataxia. Journal of neurology 1995: 242:517-21. doi 10.1007/bf00867423

[76] Takegoshi H and Murofushi T. Vestibular evoked myogenic potentials in patients with spinocerebellar degeneration. Acta oto-laryngologica 2000: 120:821-4. doi 10.1080/000164800750061660

[77] Takeichi N, Fukushima K, Sasaki H, Yabe I, Tashiro K and Inuyama Y. Dissociation of smooth pursuit and vestibulo-ocular reflex cancellation in SCA-6. Neurology 2000: 54:860-6. doi 10.1212/wnl.54.4.860

[78] Terao Y, Fukuda H, Tokushige S, Inomata-Terada S, Yugeta A, Hamada M, Ichikawa Y, Hanajima R and Ugawa Y. Is multiple system atrophy with cerebellar ataxia (MSA-C) like spinocerebellar ataxia and multiple system atrophy with parkinsonism (MSA-P) like Parkinson's disease? - A saccade study on pathophysiology. Clinical neurophysiology : official journal of the International Federation of Clinical Neurophysiology 2016: 127:1491-502. doi 10.1016/j.clinph.2015.07.035

[79] Terao Y, Fukuda H, Tokushige SI, Inomata-Terada S, Yugeta A, Hamada M and Ugawa Y. Distinguishing spinocerebellar ataxia with pure cerebellar manifestation from multiple system atrophy (MSA-C) through saccade profiles. Clinical neurophysiology : official journal of the International Federation of Clinical Neurophysiology 2017: 128:31-43. doi 10.1016/j.clinph.2016.10.012

[80] Velázquez-Pérez L, Rodríguez-Chanfrau J, García-Rodríguez JC, Sánchez-Cruz G, Aguilera-Rodríguez R, Rodríguez-Labrada R, Rodríguez-Díaz JC, Canales-Ochoa N, Gotay DA, Almaguer Mederos LE, Laffita Mesa JM, Porto-Verdecia M, Triana CG, Pupo NR, Batista IH, López-Hernandez OD, Polanco ID and Novas AJ. Oral zinc sulphate supplementation for six months in SCA2 patients: a randomized, double-blind, placebo-controlled trial. Neurochemical research 2011: 36:1793-800. doi 10.1007/s11064-011-0496-0

[81] Velázquez-Pérez L, Rodríguez-Labrada R, Álvarez-González L, Aguilera-Rodríguez R, Álvarez Sánchez M, Canales-Ochoa N, Galicia Polo L, Haro-Valencia R, Medrano-Montero J, Vázquez-Mojena Y, Peña-Acosta A, Estupiñán-Rodríguez A and Rodríguez Pupo N. Lisuride reduces involuntary periodic leg movements in spinocerebellar ataxia type 2 patients. Cerebellum (London, England) 2012: 11:1051-6. doi 10.1007/s12311-012-0382-6

[82] Velázquez-Pérez L, Rodríguez-Labrada R, Cruz-Rivas EM, Fernández-Ruiz J, Vaca-Palomares I, Lilia-Campins J, Cisneros B, Peña-Acosta A, Vázquez-Mojena Y, Diaz R, Magaña-Aguirre JJ, Cruz-Mariño T, Estupiñán-Rodríguez A, Laffita-Mesa JM, González-Piña R, Canales-Ochoa N and González-Zaldivar Y. Comprehensive study of early features in spinocerebellar ataxia 2: delineating the prodromal stage of the disease. Cerebellum (London, England) 2014: 13:568-79. doi 10.1007/s12311-014-0574-3

[83] Velázquez-Pérez L, Seifried C, Abele M, Wirjatijasa F, Rodríguez-Labrada R, Santos-Falcón N, Sánchez-Cruz G, Almaguer-Mederos L, Tejeda R, Canales-Ochoa N, Fetter M, Ziemann U, Klockgether T, Medrano-Montero J, Rodríguez-Díaz J, Laffita-Mesa JM and Auburger G. Saccade velocity is reduced in presymptomatic spinocerebellar ataxia type 2. Clinical neurophysiology : official journal of the International Federation of Clinical Neurophysiology 2009: 120:632-5. doi 10.1016/j.clinph.2008.12.040

[84] Walterfang M, Abel LA, Desmond P, Fahey MC, Bowman EA and Velakoulis D. Cerebellar volume correlates with saccadic gain and ataxia in adult Niemann-Pick type C. Molecular genetics and metabolism 2013: 108:85-9. doi 10.1016/j.ymgme.2012.11.009

[85] Walterfang M, Macfarlane MD, Looi JC, Abel L, Bowman E, Fahey MC, Desmond P and Velakoulis D. Pontine-to-midbrain ratio indexes ocular-motor function and illness stage in adult Niemann-Pick disease type C. European journal of neurology 2012: 19:462-7. doi 10.1111/j.1468-1331.2011.03545.x

[86] Wessel K, Moschner C, Wandinger KP, Kömpf D and Heide W. Oculomotor testing in the differential diagnosis of degenerative ataxic disorders. Archives of neurology 1998: 55:949-56. doi 10.1001/archneur.55.7.949

[87] Wiest G, Tian JR, Baloh RW, Crane BT and Demer JL. Otolith function in cerebellar ataxia due to mutations in the calcium channel gene CACNA1A. Brain : a journal of neurology 2001: 124:2407-16. doi 10.1093/brain/124.12.2407

[88] Wu C, Chen DB, Feng L, Zhou XX, Zhang JW, You HJ, Liang XL, Pei Z and Li XH. Oculomotor deficits in spinocerebellar ataxia type 3: Potential biomarkers of preclinical detection and disease progression. CNS Neurosci Ther 2017: 23:321-8. doi 10.1111/cns.12676

[89] Yue Q, Jen JC, Nelson SF and Baloh RW. Progressive ataxia due to a missense mutation in a calcium-channel gene. American journal of human genetics 1997: 61:1078-87. doi 10.1086/301613

[90] Zee DS, Yee RD, Cogan DG, Robinson DA and Engel WK. Ocular motor abnormalities in hereditary cerebellar ataxia. Brain : a journal of neurology 1976: 99:207-34. doi 10.1093/brain/99.2.207

[91] Moreno-Ajona D, Álvarez-Gómez L, Manrique-Huarte R, Rivas E, Martínez-Vila E and Pérez-Fernández N. VEMPs and Dysautonomia Assessment in Definite Cerebellar Ataxia, Neuropathy, Vestibular Areflexia Syndrome (CANVAS): a Case Series Study. Cerebellum (London, England) 2019. doi 10.1007/s12311-019-01061-1

[92] Szmulewicz DJ, Waterston JA, MacDougall HG, Mossman S, Chancellor AM, McLean CA, Merchant S, Patrikios P, Halmagyi GM and Storey E. Cerebellar ataxia, neuropathy, vestibular areflexia syndrome (CANVAS): a review of the clinical features and video-oculographic diagnosis. Annals of the New York Academy of Sciences 2011: 1233:139-47. doi 10.1111/j.1749-6632.2011.06158.x

[93] Yacovino DA, Zanotti E and Hain TC. Is Cerebellar Ataxia, Neuropathy, and Vestibular Areflexia Syndrome (CANVAS) a Vestibular Ganglionopathy? The journal of international advanced otology 2019: 15:304-8. doi 10.5152/iao.2019.7068

[94] Rey-Martinez J, Batuecas-Caletrio A, Matino E, Trinidad-Ruiz G, Altuna X and Perez-Fernandez N. Mathematical Methods for Measuring the Visually Enhanced Vestibulo-Ocular Reflex and Preliminary Results from Healthy Subjects and Patient Groups. Frontiers in neurology 2018: 9:69. doi 10.3389/fneur.2018.00069
